# Parabrachial *Calca* neurons drive nociplasticity

**DOI:** 10.1016/j.celrep.2024.114057

**Published:** 2024-04-06

**Authors:** Logan F. Condon, Ying Yu, Sekun Park, Feng Cao, Jordan L. Pauli, Tyler S. Nelson, Richard D. Palmiter

**Affiliations:** 1 Howard Hughes Medical Institute, University of Washington, Seattle, WA 98195, USA; 2 Departments of Biochemistry and Genome Sciences, University of Washington, Seattle, WA 98195, USA; 3 Graduate Program in Neuroscience, University of Washington, Seattle, WA 98195, USA; 4 Medical Scientist Training Program, University of Washington, Seattle, WA 98195, USA; 5 Department of Pharmacology and Therapeutics, College of Medicine, University of Florida, Gainesville, FL 32610, USA; 6 Lead contact

## Abstract

Pain that persists beyond the time required for tissue healing and pain that arises in the absence of tissue injury, collectively referred to as nociplastic pain, are poorly understood phenomena mediated by plasticity within the central nervous system. The parabrachial nucleus (PBN) is a hub that relays aversive sensory information and appears to play a role in nociplasticity. Here, by preventing PBN *Calca* neurons from releasing neurotransmitters, we demonstrate that activation of *Calca* neurons is necessary for the manifestation and maintenance of chronic pain. Additionally, by directly stimulating *Calca* neurons, we demonstrate that *Calca* neuron activity is sufficient to drive nociplasticity. Aversive stimuli of multiple sensory modalities, such as exposure to nitroglycerin, cisplatin, or lithium chloride, can drive nociplasticity in a *Calca*-neuron-dependent manner. Aversive events drive nociplasticity in *Calca* neurons in the form of increased activity and excitability; however, neuroplasticity also appears to occur in downstream circuitry.

## In brief

Condon et al. demonstrate that activity within parabrachial *Calca* neurons is both necessary for the manifestation of chronic neuropathic pain and sufficient to induce nociplasticity. Chronic activation of parabrachial *Calca* neurons via aversive stimuli or direct exogenous stimulation increases the intrinsic excitability of *Calca* neurons.

## Graphical abstract

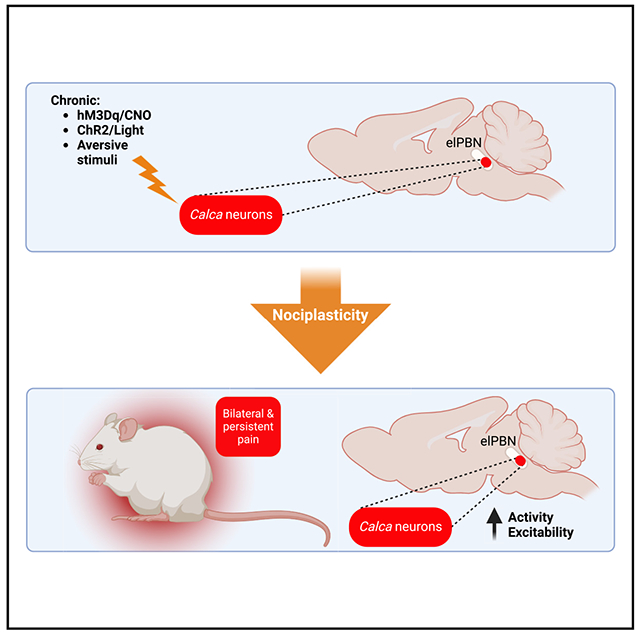

## INTRODUCTION

Pain is an evolutionarily adaptive sensory modality that signals tissue injury and triggers defensive behavioral responses. However, changes in the neural circuitry underlying pain sensation can drive pain in the absence of tissue injury, a debilitating condition referred to as nociplastic pain.^1,2^ While nociplastic pain can occur independently, it often presents as part of a mixed-pain pathophysiology, arising in parallel with chronic nociceptive or neuropathic pain.^1–4^ This suggests that persistent pain experience can drive changes in pain perception and produce a generalized pain state. Up to one in five people^4–6^ are affected by a chronic pain experience, and a nociplastic component is estimated to be present in 25%–75% of chronic pain cases.^1,7^ The neural circuitry and neuroplasticity underlying nociplastic pain are not well understood. Interrogating the pathophysiology of this widespread condition is necessary to develop therapeutic interventions that target the mechanistic underpinnings of nociplastic pain, not just the presenting symptoms.

Nociceptive signaling from the spinal cord, trigeminal and vagal nerves, and area postrema can activate parabrachial nucleus (PBN) neurons,^8–11^ which relay those signals to multiple forebrain regions.^12–17^ The PBN is composed of approximately a dozen molecularly defined glutamatergic neuron subtypes and a minor population of GABAergic neurons.^12^ Non-specific activation of the PBN glutamatergic neurons can elicit painful phenotypes, such as allodynia, while inhibition of those neurons or activation of the GABAergic neurons can inhibit painful phenotypes.^14,18,19,20^ The roles that molecularly defined subsets of PBN neurons contribute to pain sensation is also being explored. For example, chemogenetic activation of PBN *Tac1*-expressing neurons elicits escape-like behaviors,^21,22^ whereas chemogenetic or optogenetic activation of *Tacr1*-expressing neurons elicits coping responses to painful stimuli.^13,22–24^ We and others have shown that PBN *Calca* neurons are activated by aversive stimuli spanning several modalities, including some that are considered painful; e.g., foot shock, tail pinch, and formalin- or Freund adjuvant-induced inflammation.^25–29^

Chemogenetic activation of *Calca* neurons, which reside in the external lateral PBN (elPBN),^12^ promotes anorexia, adipsia, and escape-like behaviors,^21,30^ whereas more robust stimulation with optogenetics can induce freezing, bradycardia, and fear-like behaviors.^28,31^ Most assays have examined effects of transient activation, but chronic activation can promote severe anorexia,^30^ and the neurological effects of cancer cachexia have been shown to depend on activation of PBN *Calca* neurons.^26^ We show here that activation of *Calca* neurons is necessary to establish allodynia after nerve ligation. Additionally, chronic activation of these neurons is sufficient to instate chronic pain phenotypes, suggesting that the activity of *Calca* neurons drives nociplasticity.

## RESULTS

### Neuropathic pain activates parabrachial *Calca* neurons and drives persistent changes in their excitability

**Figure 1.**
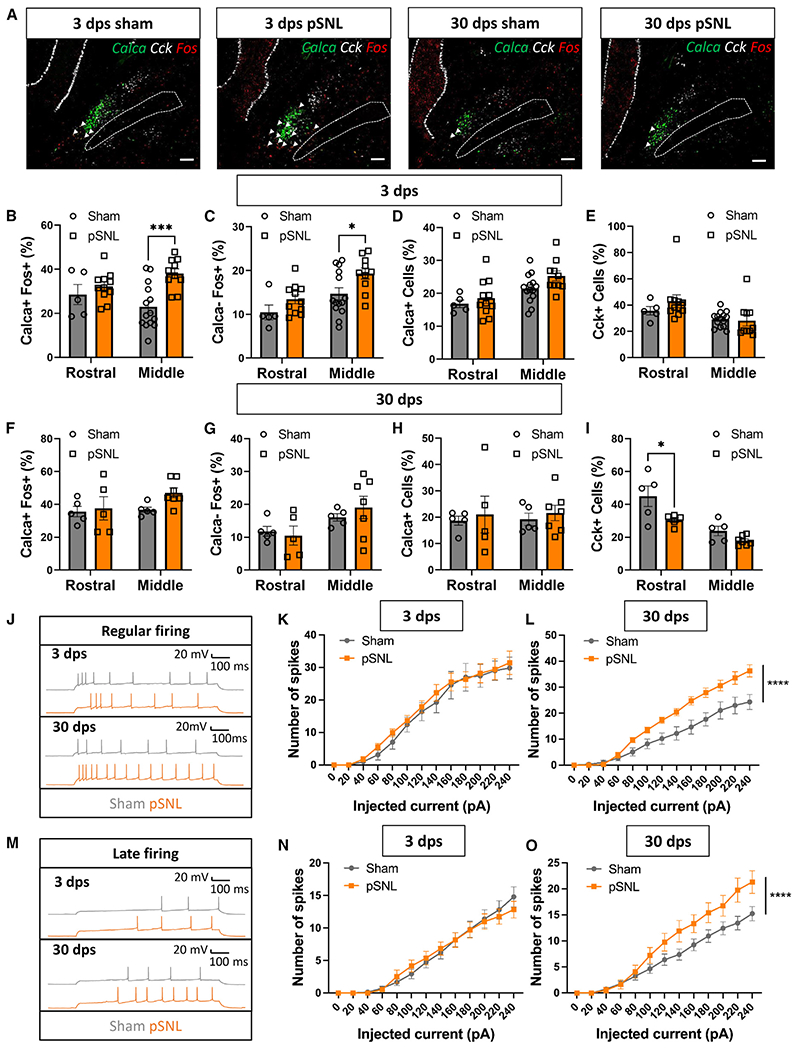
Neuropathic pain activates parabrachial *Calca* neurons and drives persistent changes in their excitability (A) Representative images from RNAscope *in situ* hybridization on tissue slices collected 3 or 30 days post sham or pSNL surgery with probes targeting *Calca* (green), *Fos* (red), and *Cck* (white). Scale bars, 100 μm. A dotted line marks the superior cerebellar peduncle (SCP); anterior-posterior bregma level = −5.1. (B) pSNL increased *Fos* mRNA in *Calca* neurons in the middle, but not the rostral, PBN at 3 dps. (C) pSNL increased *Fos* mRNA in non-*Calca* neurons in the middle, but not the rostral, PBN at 3 dps. (D) pSNL did not change the number of *Calca*-positive neurons in the rostral or middle PBN at 3 dps. (E) pSNL did not change the number of *Cck*-positive cells in the rostral or middle PBN at 3 dps. (B–E) Rostral sham, *n* = 5; middle sham, *n* = 14; rostral pSNL, *n* = 11; middle pSNL, *n* = 10. (F) pSNL did not drive an increase in the expression of *Fos* mRNA in *Calca* neurons at 30 dps. (G) pSNL did not drive an increase in the expression of *Fos* mRNA in non-*Calca* neurons at 30 dps. (H) pSNL did not change the number of *Calca*-positive neurons in the rostral or middle PBN at 30 dps. (I) pSNL decreased the number of *Cck*-positive neurons in the rostral, but not the middle, PBN at 30 dps. (F–I) Rostral sham, *n* = 5; middle sham, *n* = 5; rostral pSNL, *n* = 5; and middle pSNL, *n* = 7. (B–I) Significance was tested by ANOVA with multiple comparisons. *p < 0.05, ***p < 0.001. Error bars, SEM. (J) Representative traces showing regularly firing *Calca* neurons 3 and 30 days post sham or pSNL surgery. (K) pSNL 3 days prior to electrophysiology did not change the number of spikes elicited by current injection in the regularly firing population. Sham, *n* = 3 animals, 7 neurons; pSNL treated, *n* = 3 animals, 13 neurons. (L) pSNL 30 days prior to electrophysiology increased the number of spikes elicited by current injection in the regularly firing population. Sham, *n* = 4 animals, 9 neurons; pSNL treated, *n* = 4 animals, 9 neurons. (K and L) 3 dps sham and pSNL biological replicates, *n* = 3. 3 dps sham technical replicates, *n* = 7 and pSNL technical replicates, *n* = 13. 30 dps sham and pSNL biological replicates, *n* = 4. 30 dps sham technical replicates, *n* = 9 and pSNL technical replicates, n = 9. Significance was measured by ANOVA. ****p < 0.0001. Error bars, SEM. (M) Representative traces showing late-firing *Calca* neurons 3 and 30 days post sham or pSNL surgery. (N) pSNL 3 days prior to electrophysiology did not change the number of spikes elicited by current injection in the late-firing population. Sham, *n* = 3 animals, 15 neurons; pSNL treated, *n* = 3 animals, 13 neurons. (O) pSNL 30 days prior to electrophysiology increased the number of spikes elicited by current injection in the late-firing population. Sham, *n* = 4 animals, 20 neurons; pSNL treated, *n* = 4 animals, 9 neurons. (N and O) 3 dps sham and pSNL biological replicates, *n* = 3. 3 dps sham technical replicates, *n* = 15 and pSNL technical replicates, *n* = 13. 30 dps sham and pSNL biological replicates, *n* = 4. 30 dps sham technical replicates, *n* = 20 and pSNL technical replicates, *n* = 9. Significance was measured by ANOVA. ****p < 0.0001. Error bars, SEM.

Chronic pain was modeled using unilateral, partial sciatic nerve ligation (pSNL),^32^ and the presence of neuropathic allodynia was confirmed using the von Frey tactile-sensitivity assay. pSNL produced a significant reduction in the paw withdrawal threshold both ipsilateral and contralateral to the site of nerve injury (Figure S1A), consistent with previous findings of bilateral tactile hypersensitivity after nerve injury.^33–36^ PBN *Calca* neurons are known to act as a primary relay for brief pain signals;^25,28,37^ however, it was not known whether they are involved in the experience of chronic pain. To determine whether they are activated in chronic pain, we performed RNA-scope *in situ* hybridization on sham- or pSNL-operated mice using probes for *Calca, Cck*, and the immediate-early gene *Fos*, a proxy for neuronal activity^38^ (Figure 1A). We included *Cck* because it has been shown to be reduced 30 days following pSNL^39^ and could serve as a positive control. Three days following pSNL, we observed a significant increase in the colocalization of *Fos* and *Calca* mRNA in the PBN of pSNL-treated animals in the middle region of the PBN (bregma, −5.15 to −5.35); however, this effect did not persist at 30 days post surgery (dps) (Figures 1B and 1F). There was no difference in the colocalization of *Calca* and *Fos* at either time point when comparing the ipsilateral and contralateral PBN or when comparing males and females (Figures S1B and S1C). Additionally, in the middle region of the PBN, there was a significant increase in the *Fos* induction of *Calca*-negative PBN neurons at 3 dps but not 30 dps (Figures 1C and 1G). There was no change in the expression level of *Calca* mRNA within the PBN when comparing sham- and pSNL-treated animals at either time point (Figures 1D and 1H). However, the percentage of *Cck*-positive neurons was significantly decreased at 30 dps in the rostral region of the PBN (bregma, −4.95 to −5.15) (Figures 1E and 1I), consistent with a previous finding.^39^ These data suggest that *Calca* neurons are activated by pSNL. The lack of *Fos* induction in *Calca* neurons at 30 dps does not necessarily mean that the pSNL-driven *Calca* neuron activity has waned, as *Fos* induction is a poor marker of chronic changes in neuronal activity.^40^

To determine whether the excitability of *Calca* neurons changes following pSNL, we performed patch-clamp electrophysiology. Brain slices from *Calca*^*tdTomato*^ mice that included the PBN were prepared 3 and 30 days dps, and the *Calca* neurons were injected with 800-ms pulses of current ranging from 0 to 240 pA. Quantification of the elicited spikes revealed two distinct response patterns: regular and late firing (Figures 1J and 1M). No change in excitability was observed in the 3 dps neurons relative to neurons from sham-operated animals (Figures 1K and 1N); however, both the regular- and late-firing populations were significantly more responsive to current injection at 30 dps relative to neurons from sham-operated animals (Figures 1L and 1O). Taken together, the *in situ* hybridization data and the electrophysiological data reveal that *Calca* neurons are activated by pSNL and that this activity increases the intrinsic excitability of *Calca* neurons over time.

### Parabrachial *Calca* neurons are necessary for the induction of neuropathic pain

**Figure 2.**
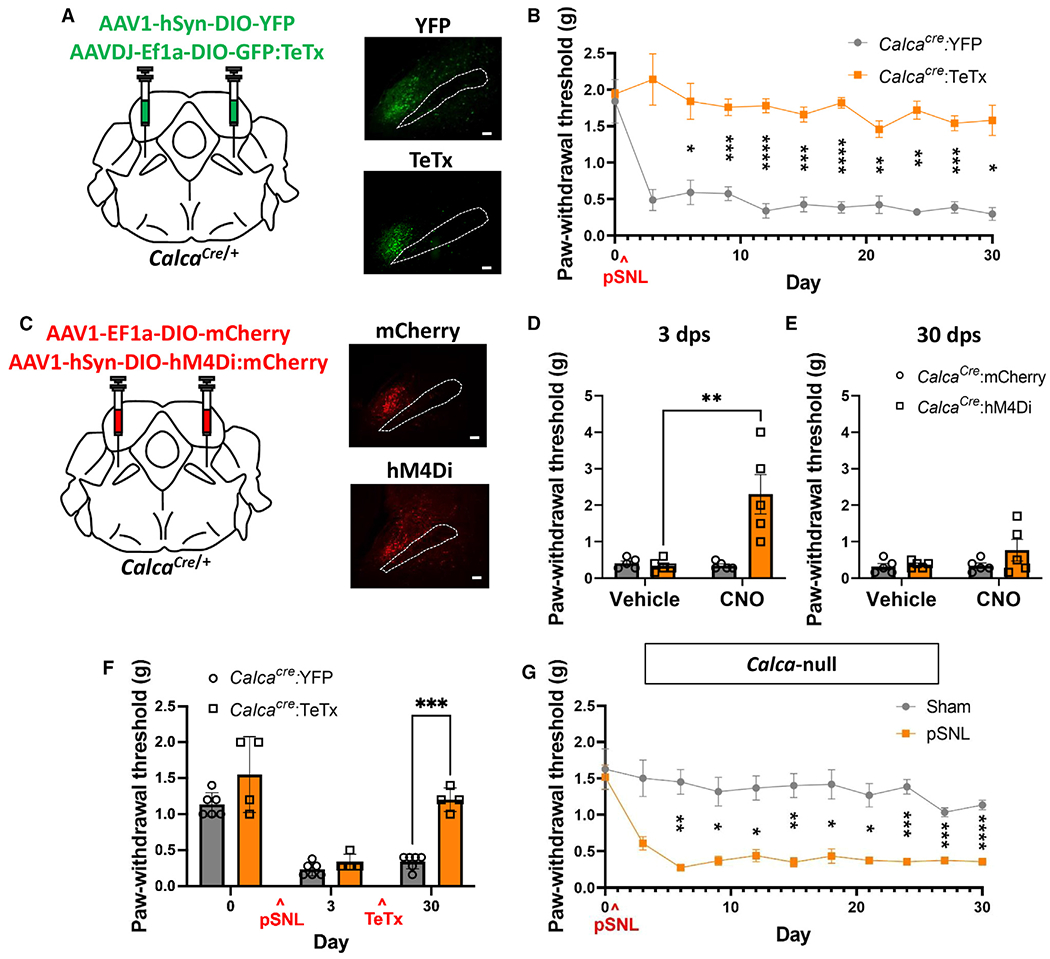
Parabrachial *Calca* neurons are necessary for the induction of neuropathic pain (A) Bilateral injections of AAV1-SYN-DIO-YFP or AAVDJ-Ef1a-DIO-GFP:TeTx into the PBN of *Calca*^*Cre*/+^ mice. Representative images show expression of YFP and TeTx. Scale bars, 100 μm. A dotted line marks the SCP; anterior-posterior bregma level = −5.1. (B) TeTx expression in PBN *Calca* neurons prevents the development of pSNL-driven allodynia, measured by von Frey assay. *Calca*^*Cre*/+^:YFP, *n* = 5 and *Calca*^*Cre*/+^: TeTx, *n* = 5. (C) Bilateral injections of AAV1-SYN-DIO-mCherry or AAV1-CBA-DIO-hM4Di:mCherry into the PBN of *Calca*^*Cre*/+^ mice. Representative images show expression of mCherry and hM4Di:mCherry. Scale bars, 100 μm. A dotted line marks the SCP; anterior-posterior bregma level = −5.1. (D) hM4Di/CNO inhibition of PBN *Calca* neurons ameliorates pSNL-driven allodynia at 3 dps, measured by von Frey assay. *Calca*^*Cre*/+^:mCherry, *n* = 5 and *Calca*^*Cre*/+^: hM4Di, *n* = 5. (E) hM4Di/CNO inhibition of PBN *Calca* neurons did not ameliorate pSNL-driven allodynia at 30 dps, measured by von Frey assay. *Calca*^*Cre*/+^:mCherry, *n* = 5 and *Calca*^*Cre*/+^:hM4Di, *n* = 5. (F) Bilateral injections of AAV1-SYN-DIO-YFP or AAVDJ-Ef1a-DIO-GFP:TeTx into the PBN of *Calca*^*Cre*/+^ mice 14 days after pSNL ameliorated established allodynia, measured by von Frey assay. *Calca*^*Cre*/+^:YFP, *n* = 6 and *Calca*^*Cre*/+^:TeTx, *n* = 4. (G) pSNL produced allodynia in *Calca*-null mice, measured by von Frey assay. (B and D–G) Significance was tested by ANOVA with multiple comparisons. *p < 0.05, **p < 0.01, ***p < 0.001, ****p < 0.0001. Error bars, SEM.

Inhibition of PBN neurons prevents the manifestation of neuropathic injury-driven allodynia;^19,20,34^ however, this phenomenon has never been associated with molecularly defined neurons within the PBN. PBN *Calca* neurons were attractive candidates because they are activated by pSNL and many other aversive stimuli.^25,29,37^ To assess the necessity of *Calca* neurons in the manifestation pSNL-driven chronic pain, we injected an AAV expressing Cre-dependent tetanus toxin light chain (AAV-DIO-GFP:TeTx) bilaterally into the PBN of *Calca*^*Cre/*+^ mice (Figure 2A) before performing pSNL or sham surgeries. TeTx degrades synaptobrevin, part of the cellular machinery responsible for vesicle release, thereby preventing signaling to post-synaptic cells.^41,42^ Prior to pSNL, a baseline sensitivity to the von Frey (mechanical allodynia) assay was obtained. TeTx expression did not affect baseline paw withdrawal threshold relative to control animals injected with an AAV expressing Cre-dependent YFP; however, after pSNL, mice treated with TeTx did not develop tactile allodynia (Figure 2B). Control animals developed allodynia as expected (Figure 2B). These data suggest that *Calca* neurons are necessary for the manifestation of allodynia in this neuropathic pain model.

We then asked whether there was a critical window following neuropathic injury during which the activity of *Calca* neurons is necessary for the manifestation of allodynia. We first bilaterally expressed Cre-dependent hM4Di (AAV-DIO-hM4Di:YFP), an inhibitory designer receptor activated by clozapine-N-oxide (CNO) ligand, in the PBN of *Calca*^*Cre/*+^ mice (Figure 2C). Tactile sensitivity was assessed at baseline using the von Frey assay, and then all animals received unilateral pSNL. The same assay was repeated at 3 dps, first following saline injection (0.9%, intraperitoneally [i.p.]) and then following CNO injection (5 mg/kg, i.p.). This sequence was performed again at 30 dps. Three days following pSNL, all mice developed tactile allodynia, and CNO-mediated inhibition of *Calca* neuron returned paw withdrawal thresholds to baseline levels (Figure 2D). At 30 dps, the allodynia was still apparent, but readministering CNO did not ameliorate pSNL-driven allodynia (Figure 2E). After observing that this transient inhibition of *Calca* neurons became ineffective at 30 dps, we explored whether chronically silencing *Calca* neurons would impact established allodynia. This was accomplished by bilaterally injecting virally expressed Cre-dependent TeTx into the PBN of *Calca*^*Cre/*+^ animals 14 days after inducing allodynia via pSNL. At 3 dps, allodynia was present, and it persisted at 30 dps in the control mice (Figure 2F). However, at 30 dps, the paw withdrawal threshold of TeTx-treated animals had returned to the normal range (Figure 2F). Given that transient inhibition of *Calca* neurons did not ameliorate chronically established allodynia, but chronic silencing of *Calca* neurons did, it is likely that neuroplasticity occurs downstream of *Calca* neurons and that the activity of *Calca* neurons is necessary for the maintenance of this downstream pro-nociceptive neuroplasticity.

After observing that activation of PBN *Calca* neurons is necessary for the manifestation of chronic pain following neuropathic injury, we explored whether neuropeptide expression from the *Calca* gene is necessary for the development of allodynia following pSNL. For these experiments, we used homozygous *Calca*^*Cre/Cre*^ mice because homozygosity precludes expression of calcitonin gene-related protein (CGRP).^27,43^ pSNL-induced nerve injury in *Calca*^*Cre/Cre*^ mice still produced persistent allodynia (Figure 2G). In line with previous findings demonstrating that the *Calca* gene is dispensable for acute pain sensation,^44^ these data indicate that CGRP is not necessary to drive allodynia in this neuropathic pain model. However, it is worth noting that loss of CGRP alleviates pain in arthritis, formalin, and bladder pain models.^43,45,46^

### Activation of parabrachial *Calca* neurons is sufficient to drive nociplasticity

Chronic stimulation of all excitatory neurons in the PBN can produce a persistent state of allodynia in the absence of tissue injury,^20^ a nociplastic effect. About 85% of PBN neurons are glutamatergic,^12,46^ including molecularly defined neurons that mediate different and sometimes opposing behavioral effects.^12,14,21,27,30,31,46^ We repeated the experiment of Sun et al.^20^ by bilaterally injecting AAV carrying a Cre-dependent, excitatory designer receptor activated by CNO (AAV-DIO-hM3Dq:mCherry) into the PBN of *Slc17a6*^*Cre/*+^ mice (*Slc17a6* encodes Vglut2 [vesicular glutamate transporter 2]). Daily treatment with CNO (1 mg/kg, i.p., 7 days) produced allodynia that developed following the first CNO injection and lasted many days after the last CNO injection, a sign of nociplasticity (Figure S2A). We replicated the results of Sun et al.,^20^ who measured mechanical sensitivity 23 h after each CNO injection; however, when von Frey sensitivity was measured 2 h after each CNO injection, it produced a remarkable analgesic effect that dissipated by 23 h, revealing allodynia (Figures S2A and S2B). The hM3Dq/CNO-driven analgesia in *Slc17a6*^*Cre/*+^ mice is similar in both scale and transience to the effect of stimulating PBN *Oprm1* neurons, a subset of the greater *Slc17a6* population,^12,21^ using the same stimulation strategy (Figure S2C). Consistent with previous studies,^47^ these data show that the PBN *Oprm1* subpopulation can mediate the analgesic effect of stimulating all PBN *Slc17a6* neurons. These findings demonstrate the functional heterogeneity of excitatory neurons within the PBN and are consistent with the molecular heterogeneity of this brain region.^12^

**Figure 3.**
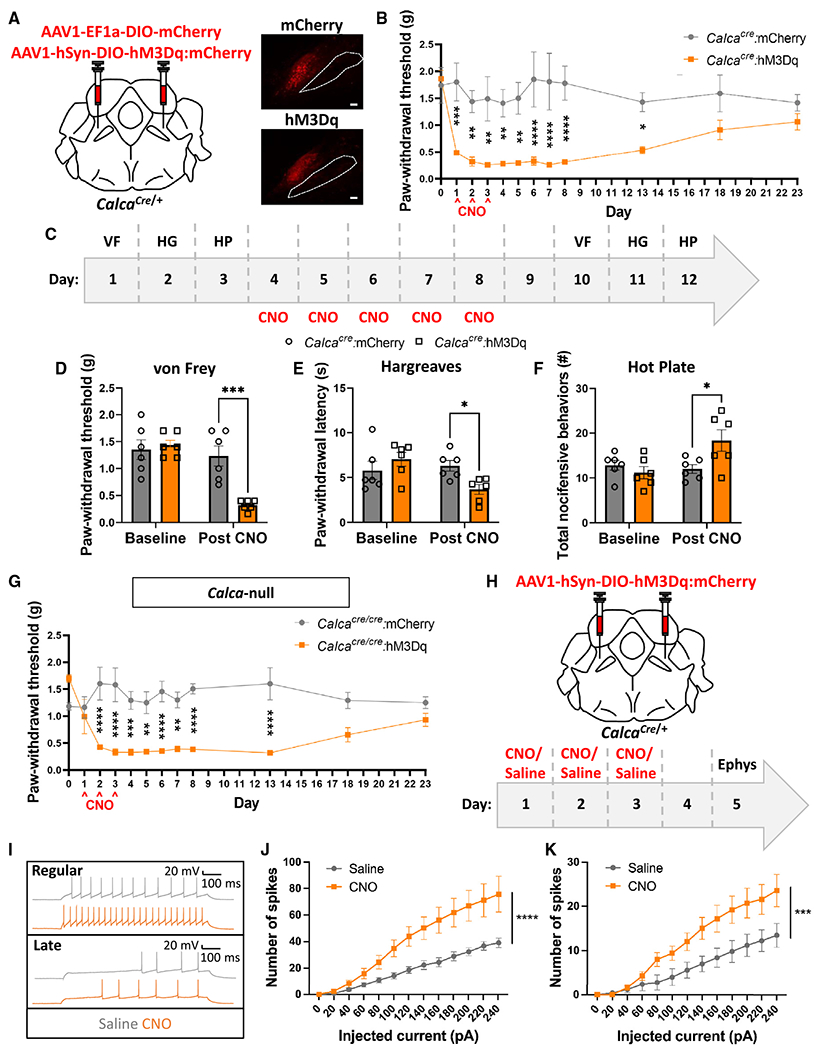
Activation of parabrachial *Calca* neurons is sufficient to drive nociplasticity (A) Bilateral injections of AAV1-Ef1a-DIO-mCherry or AAV1-hSyn-DIO-hM3Dq:mCherry into the PBN of *Calca*^*cre*/+^ mice. Representative images show expression of mCherry and hM3Dq. Scale bars, 100 μm. A dotted line marks the SCP; anterior-posterior bregma level = −5.1. (B) 3 days of CNO injection (1 mg/kg, i.p.) resulted in persistent allodynia, measured by von Frey assay. *Calca*^*cre*/+^:mCherry, *n* = 5 and *Calca*^*cre*/+^:hM3Dq, *n* = 7. (C) Behavior timeline for von Frey (VF), Hargreave’s (HG), and hot plate (HP) assays before and after 5 consecutive days of CNO injection. *Calca*^*Cre*/+^:mCherry, *n* = 6 and *Calca*^*cre*/+^:hM3Dq, *n* = 6. (D) 5 days of CNO injection decreased the paw withdrawal threshold, measured by VF assay, which persisted after the last CNO injection. (E) 5 days of CNO injection decreased the paw withdrawal latency, measured by HG assay, which persisted after the last CNO injection. (F) 5 days of CNO injection increased nocifensive behaviors, measured by HP assay, which persisted after the last CNO injection. (G) 3 days of CNO injection (1 mg/kg, i.p.) resulted in persistent allodynia in *Calca*-null mice, measured by VF assay. *Calca^Cre/Cre^*:mCherry, *n* = 6 and *Calca^Cre/Cre^*:hM3Dq, *n* = 6. (B and D–G) Significance was tested by ANOVA with multiple comparisons. *p < 0.05, **p < 0.01, ***p < 0.001, ****p < 0.0001. Error bars, SEM. (H) Bilateral injections of AAV1-SYN-DIO-hM3Dq:mCherry into the PBN of *Calca*^*cre*/+^ mice followed by 3 days of CNO or saline injection and 48 h of no stimulation prior to electrophysiology. (I) Representative traces showing regularly and late-firing *Calca* neurons. (J) 3 days of CNO injection (i.p.) prior to electrophysiology resulted in an increase in the number of spikes elicited by current injection in the regularly firing population. Saline treated, *n* = 3 animals, 14 neurons; CNO treated, *n* = 3 animals, 11 neurons. (K) 3 days of CNO injection (i.p.) prior to electrophysiology resulted in an increase in the number of spikes elicited by current injection in the late-firing population. Saline treated, *n* = 3 animals, 5 neurons; CNO treated, *n* = 3 animals, 11 neurons. (J and K) Significance was measured by ANOVA. ***p < 0.001, ****p < 0.0001. Error bars, SEM.

Given that PBN *Calca* neurons are necessary for the manifestation of neuropathic injury-driven allodynia, we hypothesized that activation of PBN *Calca* neurons might be sufficient to produce nociplasticity. To test this idea, we injected an AAV carrying Cre-dependent hM3Dq:mCherry (or just mCherry as a control) bilaterally into the PBN of *Calca*^*Cre/*+^ mice (Figure 3A). After several weeks for viral expression, 3 consecutive days of CNO delivery (1 mg/kg, i.p.) resulted in significant tactile allodynia 2 h and 23 h post injection that persisted for 10 days following the last CNO injection (Figures 3B and S2D). We noted a sexually dimorphic effect in the dissipation but not the development of allodynia (Figure S2E). Additionally, unilateral injection of AAV with Cre-dependent hM3Dq:mCherry and subsequent treatment with CNO produced bilateral allodynia (Figures S2F and S2G). To determine whether this persistent allodynic effect was, in fact, nociplasticity, not simply a learned association between the von Frey chamber and *Calca*-neuron-driven aversive sensation, we performed a set of pain assays before and after, but not during, 5 days of CNO administration (Figure 3C). Two days after the last CNO injection, we observed a significant decrease in von Frey paw withdrawal threshold, Hargreaves paw withdrawal latency, and the number of nocifensive behaviors on a hot plate (Figures 3D–3F). We also tested whether chronic activation of *Calca* neurons alters locomotor ability as well as anxiety and depression-related behaviors (Figure S3A). After 5 days of CNO or saline administration, CNO-treated animals exhibited elevated locomotor activity in the open field assay, elevated freezing in the tail suspension test relative to controls, and decreased paw withdrawal threshold (Figures S3B, S3E, and S3F). There was a trend toward a significant decrease in sucrose preference (*p* = 0.176; Figure S3G) and no significant effect in the open field center time or elevated plus maze assays (Figures S3C and S3D). The persistent allodynia and hyperalgesia observed in these assays support our conclusion that chronic activity in the PBN *Calca* population is sufficient to drive nociplasticity.

Given that activation of PBN *Calca* neurons is sufficient to drive nociplastic pain, we asked whether expression of the *Calca* gene is a necessary part of these phenomena. We performed 3 consecutive days of hM3Dq-mediated stimulation of PBN *Calca* neurons in *Calca*^*Cre/Cre*^ mice. This manipulation produced allodynia that lasted 10 days after CNO cessation (Figure 3G), like the effect observed using heterozygous *Calca*^*Cre/*+^ mice (Figure 3B). These data indicate that, while activation of PBN *Calca* neurons is sufficient to drive nociplasticity, the *Calca* gene products, including CGRP, do not play a necessary role in this model of nociplastic pain.

We established that *Calca* neuron activity is sufficient to drive nociplasticity; however, the neuronal populations exhibiting neuroplastic changes were not yet established. We performed patch-clamp electrophysiology to assess the excitability of *Calca* neurons expressing hM3Dq:mCherry following 3 days of either CNO or saline injections (Figures 3H and 3I). Forty-eight hours following the final injection, at which point all injected CNO should be metabolized,^48^ *Calca* neurons in slices were injected with 800-ms pulses of current ranging from 0 to 240 pA. Quantification of the elicited spikes once again revealed two distinct response patterns: regular and late firing (Figure 3J). Both the regular- and late-firing populations were significantly more responsive to current injection when the mice were pretreated with CNO rather than saline (Figures 3K and 3L). These data demonstrate that this direct activation of *Calca* neurons produces a persistent increase in their intrinsic excitability.

### Nociplastic allodynia scales with the duration of *Calca* neuron activation

**Figure 4.**
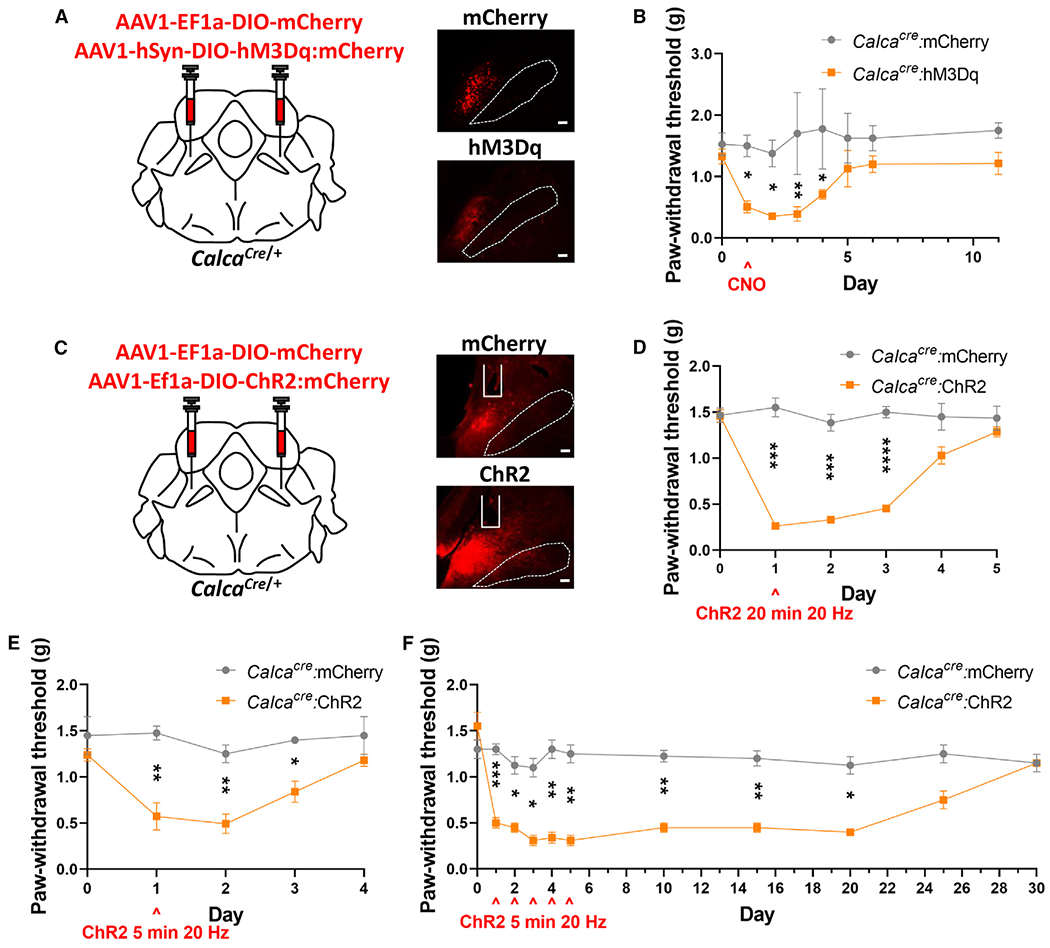
Nociplastic effect scales with the duration of *Calca* neuron activation (A) Bilateral injections of AAV1-Ef1a-DIO-mCherry or AAV1-hSyn-DIO-hM3Dq:mCherry into the PBN of *Calca*^*cre*/+^ mice. Representative images show expression of mCherry and hM3Dq. Scale bars, 100 μm. A dotted line marks the SCP; anterior-posterior bregma level = −5.1. (B) 1 day of CNO injection (1 mg/kg, i.p.) produced allodynia, measured by VF assay. *Calca*^*cre*/+^:mCherry, *n* = 4 and *Calca*^*cre*/+^:hM3Dq, *n* = 7. (C) Bilateral injections of AAV1-Ef1a-DIO-mCherry or AAV1-Ef1a-DIO-ChR2:mCherry into the PBN of *Calca*^*cre*/+^ mice. Representative images show expression of mCherry and ChR2. Scale bars, 100 μm. A dotted line marks the SCP; anterior-posterior bregma level = −5.1. (D) 20 min of 473-nm photostimulation (20 Hz, 2 s on, 2 s off) resulted in allodynia, measured by VF assay. *Calca*^*cre*/+^:mCherry, *n* = 6 and *Calca*^*cre*/+^:ChR2, *n* = 7. (E) 5 min of 473-nm photostimulation (20 Hz, 2 s on, 2 s off) resulted in allodynia, measured by VF assay. *Calca*^*cre*/+^:mCherry, *n* = 4 and *Calca*^*cre*/+^:ChR2, *n* = 5. (F) 5 days of 5-min, 473-nm photostimulation (20 Hz, 2 s on, 2 s off) resulted in allodynia, measured by VF assay. *Calca*^*cre*/+^:mCherry, *n* = 4 and *Calca*^*cre*/+^:ChR2, *n* = 4. (B and D–F) Significance was tested by ANOVA with multiple comparisons. *p < 0.05, **p < 0.01, ***p < 0.001, ****p < 0.0001. Error bars, SEM.

Seven days of stimulating *Slc17a6* neurons in the PBN produces persistent (>30 days) nociplasticity (Sun et al.^20^; Figure S2A), whereas 3 days of stimulating *Calca* neurons in the PBN produced a nociplastic effect that lasted 10 days (Figure 3B). These findings suggest that nociplasticity scales with the duration of *Calca* neuron stimulation. To further examine this phenomenon, we stimulated PBN *Calca* neurons expressing hM3Dq just once and assessed tactile allodynia until paw withdrawal thresholds returned to baseline. One day of CNO-driven stimulation of *Calca* neurons produced 4 days of allodynia (Figures 4A and 4B).

Even more acute stimulation was achieved by bilateral photostimulation of channelrhodopsin (ChR2) that was targeted to the PBN *Calca* neurons. Twenty minutes of bilateral 473-nm (20 Hz, 10 mW, 2 s “on,” 2 s “off”) light application over the *Calca* cell bodies produced allodynia within an hour and persisted for 2 days after stimulus cessation (Figures 4C and 4D). Even 5 min of bilateral stimulation produced allodynia that was weaker but lasted 2 days (Figure 4E). Additionally, 5 min of stimulation performed daily for 5 days produced allodynia that lasted 15 days after the last stimulation (Figure 4F). A single 20-min unilateral stimulation using the same parameters also produced bilateral nociplasticity (Figure S4A). Together, these data suggest that the duration, and probably the intensity, of PBN *Calca* neuron stimulation dictates the duration of the resultant nociplasticity.

### Chronic exposure to aversive stimuli drives nociplasticity across a range of sensory modalities

**Figure 5.**
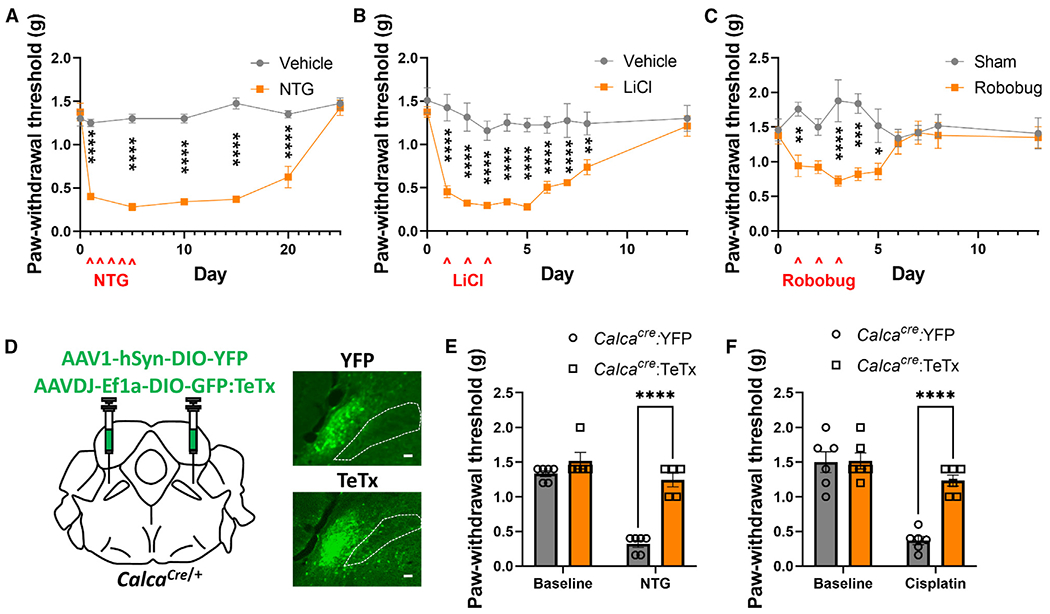
Chronic exposure to all aversive stimuli tested drives nociplasticity regardless of sensory modality (A) 5 days of NTG exposure (10 mg/kg, i.p.) produced allodynia, measured by VF assay. Vehicle, *n* = 4 and NTG, *n* = 4. (B) 3 days of LiCl exposure (0.2 M, 15 mL/kg, i.p.) produced allodynia, measured by VF assay. Vehicle, *n* = 6 and LiCl, *n* = 8. (C) 3 days of robobug chase (10 min) produced allodynia, measured by VF assay. Vehicle, *n* = 5 and robobug, *n* = 5. (D) Bilateral injections of AAV1-hSYN-DIO-YFP or AAVDJ-Ef1a-DIO-GFP:TeTx into the PBN of *Calca*^*Cre*/+^ mice. Representative images show expression of YFP and TeTx. Scale bars, 100 μm. A dotted line marks the SCP; anterior-posterior bregma level = −5.1. (E) TeTx expression in PBN *Calca* neurons prevented the development of NTG-driven allodynia, measured by VF assay. *Calca*^*Cre*/+^:YFP, *n* = 6 and *Calca*^*Cre*/+^:TeTx, *n* = 5. (F) TeTx expression in PBN *Calca* neurons prevented the development of cisplatin-driven allodynia, measured by VF assay. *Calca*^*Cre*/+^:YFP, *n* = 6 and *Calca*^*Cre*/+^:TeTx, *n* = 6. (A–C, E, and F) Significance was tested by ANOVA with multiple comparisons. *p < 0.05, **p < 0.01, ***p < 0.001, ****p < 0.0001. Error bars, SEM.

PBN *Calca* neurons respond to aversive stimuli spanning a range of sensory modalities.^25,29,30^ Given that stimulation of *Calca* neurons is sufficient to drive nociplasticity, we explored whether the induction of nociplasticity was agnostic to stimulus modality. Cisplatin chemotherapy,^49,50^ lithium chloride (LiCl)-induced visceral malaise,^27,30^ foot shock,^25^ and the threat of predation^29^ have all been shown to activate PBN *Calca* neurons. Chronic exposure to cisplatin,^49^ nitroglycerin (NTG; a model for migraine pain),^51,52^ and foot shock^53^ are known to produce persistent allodynia. Consistent with previous studies,^51^ we found that 5 days of NTG exposure (10 mg/kg, i.p.) produced persistent allodynia (Figure 5A). We also found that 3 days of injection with LiCl (0.2 M at 15 mL/kg, i.p.) produced persistent allodynia (Figure 5B). Even 3 days of exposure to a predatory threat (5-min chase daily for 3 days with a toy robotic bug) could promote mild nociplasticity, measured by the von Frey assay (Figure 5C).

NTG- and cisplatin-produced nociplasticity was prevented by prior expression of TeTx in *Calca* neurons (Figures 5D–5F). Interestingly, the photophobia that developed following NTG injection did not persist as long as the allodynia (Figure S5A). Additionally, despite pretreatment of the PBN *Calca* neurons with TeTx, photophobia still occurred following NTG injection (Figure S5B). These data suggest that chronic exposure to these aversive stimuli, spanning a range of sensory modalities, can induce nociplasticity via the activation of *Calca* neurons.

### *Calca* neurons exhibit plasticity following activation

**Figure 6.**
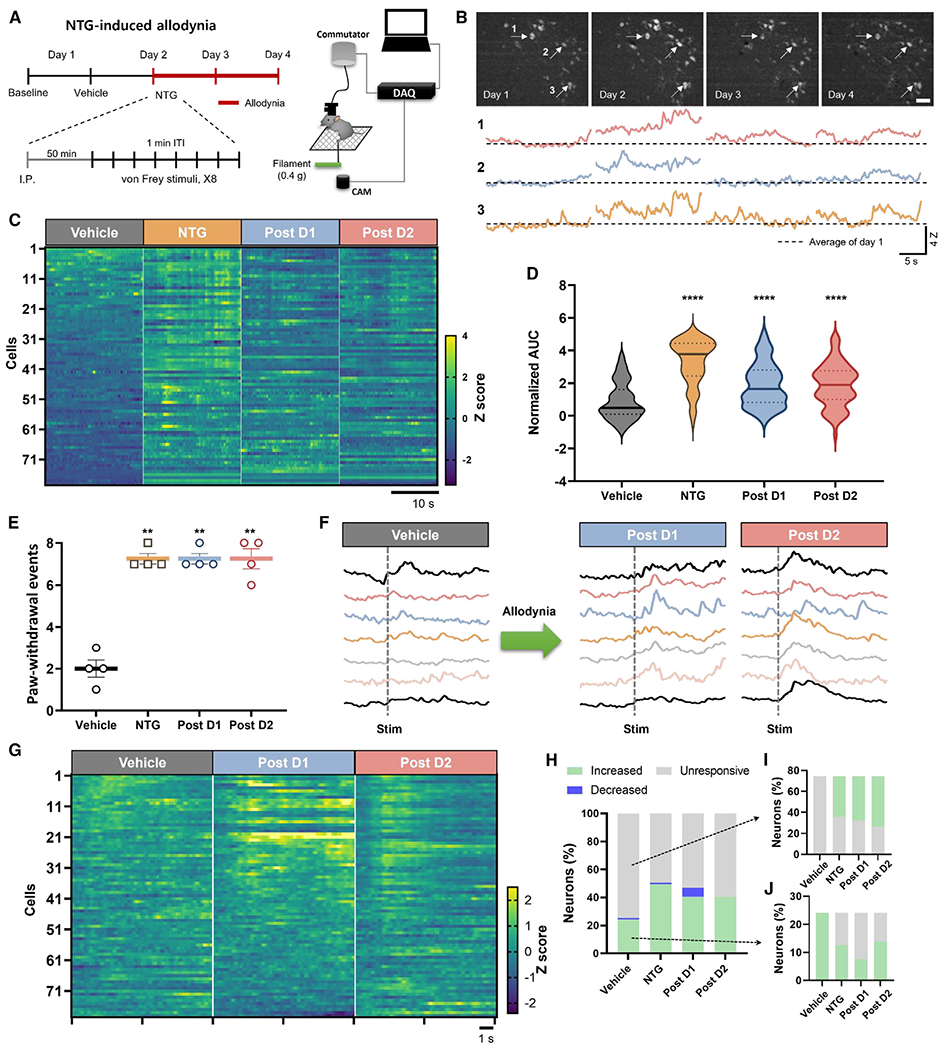
*Calca* neuron activity during the development of mechanical allodynia (A) Schematic of Ca^2+^ imaging during NTG-induced mechanical allodynia. (B) Multiday tracking of *Calca* neuron fluorescence activity. Top: field of view (FOV) of a representative animal for 4 days. Bottom: representative neural activities throughout 4 days of 3 neurons marked by arrows in FOVs. Scale bar, 50 μm. (C) NTG injection increased unstimulated neuronal activity. Elevated neuronal activity persisted 24 and 48 h post injection. Individual neurons are aligned across days in the heatmap. (D) Average calcium transient area under the curve increased following NTG injection. This increase in fluorescence activity remained elevated 24 and 48 h post injection. (E) NTG injection increased the number of times of 8 applications that mice responded to a 0.4-g VF filament. Error bars indicate mean ± SEM. (F) Representative traces of filament-evoked neural activities. (G) The number of *Calca* neurons responsive to application of a 0.4-g VF filament increased after NTG injection. The increase in responsive neurons persisted 24 and 48 h after NTG injection. (H) The percentage of *Calca* neurons responsive to application of a 0.4-g VF filament increased from 24% after vehicle injection to 49.5% after NTG injection. The percent of 0.4-g VF filament-responsive neurons remained elevated, at 40.5%, 24 and 48 h after NTG injection. (I) The majority of neurons unresponsive to 0.4-g VF filament application following vehicle injection (i.p.) became responsive to the 0.4-g filament following NTG injection (i.p.). (J) About half of the neurons responsive to 0.4-g VF filament application following vehicle injection (i.p.) became unresponsive to the 0.4-g filament following NTG injection (i.p.). (A–J) *n* = 4 animals, 79 neurons. Significance was tested by ANOVA with multiple comparisons. *p < 0.05, **p < 0.01, ***p < 0.001, ****p < 0.0001. Error bars, SEM.

NTG administration induces *Calca*-neuron-dependent, persistent allodynia, like the effect of hM3Dq/CNO-mediated stimulation, so we hypothesized that it does so by increasing the activity of *Calca* neurons. To investigate this hypothesis, we conducted Ca^2+^ imaging experiments in which we tracked the activity of individual *Calca* neurons across multiple days. To monitor the activity of *Calca* neurons in behaving animals, AAV-Ef1a-DIO-GCaMP6m was expressed, and a gradient refractive index (GRIN) lens was implanted over the PBN of *Calca*^Cre/+^ mice. After 4 weeks of recovery, a microendoscope was attached to each mouse and left in place for 4 consecutive days. This strategy ensured a stable field of view for tracking individual neurons each day. On day 1, mice received an i.p. injection of vehicle, and on day 2 they received NTG (10 mg/kg, i.p.). After each injection, mice were placed in the von Frey testing apparatus for 35 min of acclimation, followed by 5 min of baseline neuronal activity measurement and then 10 min of von Frey testing (8 filament applications with a 1-min intertrial interval). On days 3 and 4, *Calca* neurons were imaged again without further injections to determine whether NTG treatment had residual effects that could drive persistent allodynia (Figure 6A).

We tracked the same 79 neurons throughout the experiment (Figure 6B). NTG administration dramatically increased the basal fluorescence level of most of the *Calca* neurons compared with vehicle administration (Figures 6B and 6C). Notably, this elevated fluorescence activity was sustained on the 2 days following NTG injection (Figures 6B and 6C); 60% of neurons continued to exhibit elevated basal fluorescence on days 3 and 4 (Figures 6C and S6A). The number and amplitude of Ca^2+^ transients did not change across days (Figures S6B and S6C). The averaged basal fluorescence level of all 79 neurons (4 mice) increased after NTG administration and remained elevated for 2 days (Figure 6D).

We also examined whether NTG administration was accompanied by an increase in the von Frey-elicited responses of individual neurons. NTG injection increased paw withdrawal responses of all mice to a low-threshold (0.4 g) von Frey filament (Figure 6E). Some neurons that exhibited increased activity following von Frey stimuli to vehicle injection showed similar activity after NTG injection (Figure 6F), while most *Calca* neurons displayed increased fluorescence only after NTG injection (Figures 6F and 6G). On the vehicle injection day, 24% of neurons were activated by the 0.4-g von Frey filament, whereas 49.4% were activated after NTG treatment, and 40.5% responded on days 3 and 4. (Figure 6H). Analysis of individual *Calca* neurons across experimental days revealed that 56.7% of the initially unresponsive neurons became responsive after NTG, with similar values on days 3 and 4 (Figure 6I). Of the von Frey-responsive neurons on the vehicle injection day, about half lost their responsiveness following NTG injection and across the subsequent imaging sessions (Figure 6J). There were no persistent changes in the area under the curve of calcium transients, the number of calcium transients, or the amplitude of calcium transients following 0.4-g von Frey stimulation (Figures S6D–S6F). These data suggest that the primary effect of NTG treatment is the recruitment of more *Calca*-responsive neurons rather than an increase in the frequency or magnitude of their responses.

### Intrathecal injection of neuropeptide Y does not reverse hM3Dq/CNO-driven allodynia

**Figure 7.**
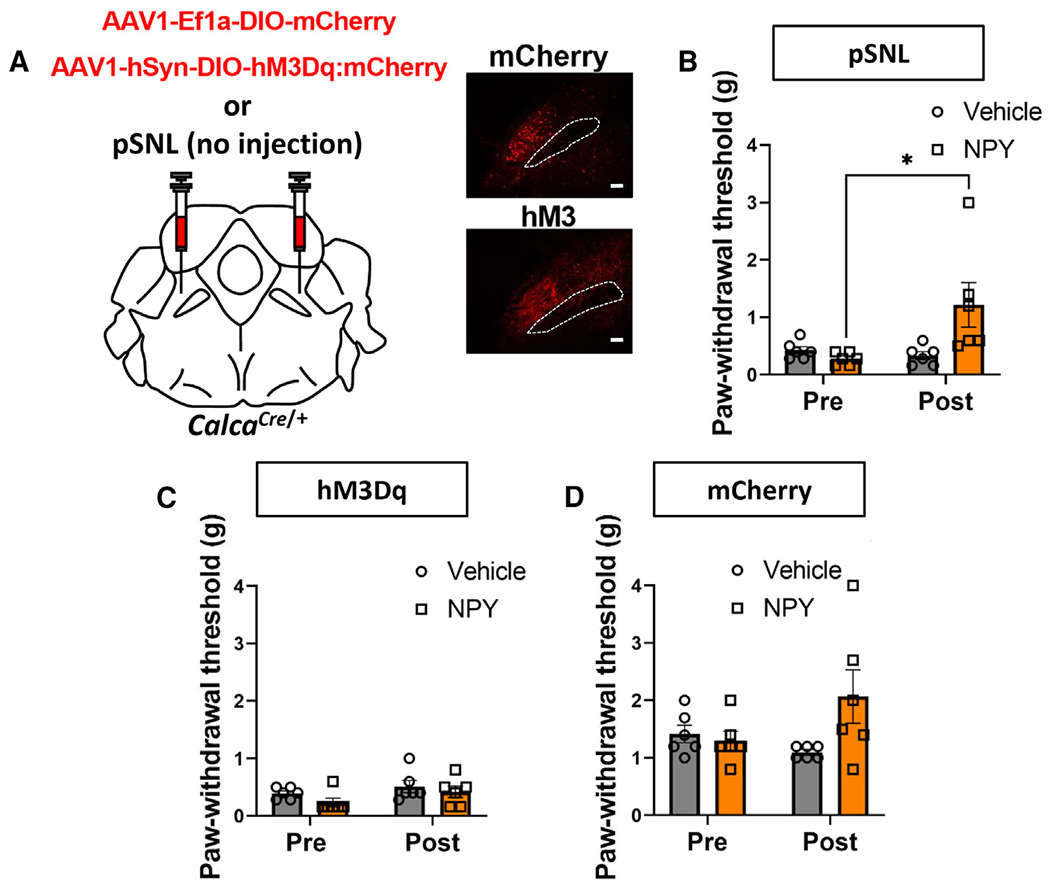
Intrathecal NPY does not reverse hM3Dq/CNO-driven allodynia (A) pSNL or bilateral injections of AAV1-Ef1a-DIO-mCherry or AAV1-hSyn-DIO-hM3Dq:mCherry into the PBN of *Calca*^*cre/*+^ mice. Representative images show expression of mCherry and hM3Dq. Scale bars, 100 μm. A dotted line marks the SCP; anterior-posterior bregma level = −5.1. (B) Intrathecal (i.t.) injection of NPY^Leu,Pro^ into pSNL animals reversed pSNL-driven allodynia. (C) I.t. injection of NPY^Leu,Pro^ into *Calca*^*Cre*^:hM3Dq animals treated with CNO for 3 days did not reverse allodynia. (D) I.t. injection of NPY^Leu,Pro^ into control *Calca*^*Cre*^:mCherry animals treated with CNO for 3 days did not affect the paw withdrawal threshold. (B–D) Significance was tested by ANOVA with multiple comparisons. *p < 0.05, **p < 0.01, ***p < 0.001, ****p < 0.0001. Error bars, SEM.

How does manipulation of neurons in the brain result in tactile allodynia, allowing gentle touch to appear painful? The allodynia that develops after sciatic nerve injury has been shown to involve plasticity in spinal dorsal horn inhibitory neurons that normally prevent (gate) low-threshold, primary afferent Aβ activity from reaching the nociceptive spinoparabrachial projection neurons, thereby allowing gentle touch to the hindpaw to promote paw withdrawal as if it was painful.^54–60^ We hypothesized that chemogenetic activation of *Calca* neurons may activate descending circuits to the spinal cord, resulting in plasticity that resembles that induced by nerve injury. Pharmacological or genetic manipulation of several different populations of excitatory neurons in the spinal cord has been shown to reverse peripheral nerve injury-induced allodynia.^54–56^ For example, intrathecal injection of the neuropeptide Y (NPY) Y1 receptor 1 agonist NPY^Leu,Pro^ has been shown to transiently reduce allodynia after sciatic nerve injury.^55,60,61,62^ Thus, we tested our hypothesis by intrathecal injection of NPY^Leu,Pro^ into mice with pSNL (as a positive control) or mice that had developed tactile allodynia after 3 days of *Calca* neuron activation with hM3Dq/CNO (Figure 7A). NPY^Leu,Pro^ injections ameliorated the allodynia produced by pSNL as expected but had no effect on the allodynia that developed after *Calca* neuron activation or on the paw withdrawal threshold of controls (Figures 7B–7D). This result indicates that the nociplastic allodynia that develops after *Calca* neuron activation does not rely on the same mechanism(s) as nociplasticity driven by pSNL.

## DISCUSSION

We demonstrated that *Calca* neuron signaling is necessary for the induction of pSNL-induced allodynia and that daily chemogenetic or optogenetic stimulation of *Calca* neurons is sufficient to induce allodynia and hyperalgesia in naive mice that persists for many days beyond the stimulation period. These chronic pain symptoms are the result of a generalized nociplasticity and not a learned association between the assays and the aversion generated by *Calca* neuron activity. Although *Calca* neuron activity is both necessary and sufficient to generate chronic pain, *Calca* gene products (i.e., CGRP) do not play a significant role in this effect. This is unexpected because CGRP signaling has been shown to be important in other pain models.^43,45,46^ While the *Calca*^*Cre/Cre*^ line that we used is a constitutive knockout of the *Calca* gene, the same line of mice was used in one of the previous studies.^43^ Therefore, it is unlikely that a compensatory developmental mechanism obscured the role that CGRP plays in neuropathic pain sensation or *Calca* neuron driven nociplastic pain.

Sun et al.^20^ reported that 7 days of chemogenetic activation of all glutamatergic neurons in the PBN could produce allodynia that lasted for many days after the final CNO injection. We obtained similar results when assaying von Frey sensitivity 23 h after each CNO injection, when CNO would have been cleared from the circulation. Remarkably, when we assayed allodynia 2 h after each CNO injection, when hM3Dq would still be activated, there was a dramatic analgesic effect. A similar effect was achieved with hM3Dq-mediated stimulation of *Oprm1* neurons. In contrast, chemogenetic activation *Calca* neurons, which represent ~15% of the glutamatergic neurons,^12^ drives allodynia at both 2 and 23 h after CNO. This result suggests that the glutamatergic population includes neurons that promote allodynia (*Calca* neurons) and those that promote analgesia (*Oprm1* neurons). Both genes are expressed in several molecularly defined clusters in the PBN.^12^ The activation of *Oprm1* neurons is aversive,^63^ so it is likely that the analgesia we observed was stress induced.^64^

*Calca*-neuron-mediated nociplasticity scales with stimulus duration. Multiple rounds of chemogenetic stimulation produced allodynia that persisted longer than a single stimulation. Likewise, a brief optogenetic stimulation produced allodynia that was less persistent than after multiple stimulations. The stimulus scaling of nociplasticity that we observed is consistent with the conclusion that *Calca* neuron activity drives nociplasticity.

Several studies, ours included, have demonstrated that unilateral nerve injury can produce bilateral pain.^26–33,65^ This phenomenon indicates that persistent pain has the capacity to induce generalized nociplasticity. Because artificial activation of *Calca* neurons is sufficient to produce generalized pain, and a wide variety of aversive situations activate *Calca* neurons, some of which are not generally considered to be painful, we predicted that many aversive experiences, especially if repeated, would have the capacity to drive nociplasticity. Chronic exposure to NTG (a model of migraine) has been shown to produce persistent allodynia,^51^ an effect that we duplicated and went on to show was dependent on activation of *Calca* neurons. Similarly, cisplatin-induced chemotherapy can produce allodynia,^49^ activate *Calca* neurons,^49,50,66^ and produce persistent allodynia that, we show, depends on *Calca* neuron activation in the PBN. We also demonstrate that prolonged visceral malaise (nausea) induced by treatment with LiCl as well as predatory simulation are sufficient to induce allodynia that may persist for many days. These findings point to a mechanism by which diverse and polymodal aversive experiences can produce nociplastic pain phenotypes via the activation of PBN *Calca* neurons. An implication of this result is that nociplastic pain may be produced by adverse life events that are distinct from somatic nerve injury. This phenomenon has been observed epidemiologically. Individuals with adverse childhood experiences are significantly more likely to develop chronic pain.^67,68^ These findings may also explain the high degree of mixed pain pathophysiology in chronic pain patients.^1^ In this circumstance, it is possible that persistent nociceptive or neuropathic pain drives nociplastic pain via the chronic activation of *Calca* neurons.

*Calca* neurons exhibit plasticity in the form of increased intrinsic excitability following 3 days of CNO stimulation of hM3Dq and an increase in the number of neurons responsive to innocuous tactile stimulation for several days after NTG exposure. These effects appear to both initiate and maintain a persistent pain state. However, our data showing that hM4Di inhibition of *Calca* neurons does not reverse allodynia at 30 days suggest that *Calca* neurons are not the only site of pronociceptive neuroplasticity in this circuit. We suggest that long-lasting allodynia involves nociplastic changes in both *Calca* neurons and the circuitry downstream of *Calca* neurons,^19,69–72^ possibly including the spinal microcircuitry. Chronic pain after sciatic nerve injury changes the microcircuitry of the dorsal horn, allowing Aβ fibers to indirectly stimulate spinoparabrachial projection neurons via a neural circuit that is inhibited under non-painful conditions.^54–60^ Intrathecal injection of NPY can reverse neuropathic pain,^55,61^ a result that we confirmed in the pSNL model. However, NPY did not ameliorate the tactile allodynia that develops after hM3Dq/CNO activation of *Calca* neurons, suggesting that neuroplasticity that promotes allodynia after pSNL is distinct from what occurs after *Calca*-mediated allodynia.

In conclusion, our study highlights the critical role of *Calca* neurons in driving nociplasticity and persistent pain states. We have shown that activation of *Calca* neurons induces allodynia and hyperalgesia that persist beyond the stimulation period, implicating a generalized nociplasticity rather than learned associations. Furthermore, our findings suggest that diverse aversive experiences can activate *Calca* neurons and contribute to the development of nociplastic pain, shedding light on the potential mechanisms underlying chronic pain conditions that extend beyond somatic nerve injury. This research underscores the complexity of pain pathophysiology and offers insights into the interplay between neural circuits and aversive life events in shaping chronic pain phenotypes.

### Limitations of the study

We identified PBN *Calca* neurons as being necessary for the manifestation of chronic neuropathic pain, and activating them was sufficient to induce physiologically and behaviorally observable nociplasticity. However, we did not identify the molecular mechanism(s) by which *Calca* neurons develop heightened excitability. The neurocircuitry leading to *Calca* neuron activation after pSNL is not established, and it is possible that non-*Calca* neurons in the PBN participate in the development of neuroplasticity. Additionally, we observed that transient inhibition of *Calca* neurons is not sufficient to ameliorate established allodynia, suggesting that pronociceptive neuroplasticity occurs downstream of *Calca* neurons that maintains nociplastic pain. Because intrathecal NPY^Leu,Pro^ injections did not ameliorate *Calca* neuron-driven allodynia, the spinal cord circuitry by which light-touch information carried by Aβ fibers reaches the PBN remains unknown. Consequently, the neurocircuitry linking *Calca* neuron activity to allodynia needs to be established. Previous results demonstrating allodynia after CNS neurons activation suggest that the central nucleus of the amygdala, basal forebrain, and rostroventral medulla are likely involved.^19,69–71,73^ Finally, we demonstrated the lack of a sex difference in the induction of *Fos* by *Calca* neurons following pSNL and a modest sex difference in the recovery from allodynia following hM3Dq/CNO-mediated excitation of *Calca* neurons, but we did not assess sexually dimorphic behaviors in any other experiments.

## STAR★METHODS

### RESOURCE AVAILABILITY

#### Lead contact

Further information and request for resources and reagents should be directed to and will be fulfilled by Richard Palmiter (Palmiter@UW.edu), the lead contact.

#### Materials availability

This study did not generate any new reagents or behavioral systems.

#### Data and code availability

- Data will be made available upon reasonable request to the corresponding author.
- All original code has been deposited at GitHub and is publicly available at https://doi.org/10.5281/zenodo.10725342.
- Any additional information required to reanalyze the data reported in the manuscript is available from the lead contact upon request.

### EXPERIMENTAL MODEL AND STUDY PARTICIPANT DETAILS

#### Mice

All experiments followed protocols approved by the Institutional Animal Care and Use Committee at the University of Washington and were in accordance with the National Institute of Health guidelines for animal research. Experiments on wild-type animals used C57BL/6 mice. Most experiments used heterozygous *Calca*^*Cre*/+^ or homozygous *Calca^Cre/Cre^* mice on a C57BL/6 genetic background were generated and maintained as described.^30^ One set of experiments used *Slc17a6*^*Cre*/+^ or *Oprm1* ^*Cre*/+^ mice. Another set used *Calca^tdTomato^* mice, which have been validated.^12^ Male and female animals were 7–9 weeks of age at the onset of all experiments and no more than 18 weeks by their experimental endpoint. Prior to surgical manipulation, animals were group housed, had access to food and water *ad libitum*, and were kept on a 12-h, light-dark cycle at 22°C. After surgical manipulation animals were singly housed, all other housing parameters remained consistent. All experiments included 4–10 experimental animals (e.g., transduced with viruses allowing Cre-dependent expression of hM3Dq, hM4Di, ChR2, TeTx) and 4–7 control animals (e.g., transduced with mCherry or YFP) to account for potential variation between experimental sessions. Animals from the same litter were randomly assigned to experimental or control groups. All experiments were performed blind, except the *Oprm1*^*Cre*/+^ experiment. Individual cohorts of mice were used for all experiments except those mentioned below. The same cohort of *Calca*^*Cre*/+^:TeTx animals was used in the NTG and cisplatin experiments with 1 week of recovery between NTG and cisplatin injections. The same cohort of *Calca*^*Cre*/+^:ChR2 animals was used for all ChR2 experiments with 1 week of recovery between the 1-day ChR2 stimulation and the 5-day ChR2 stimulation experiment. Viral transduction was assessed at the end of each experiment histologically and only mice with correct expression were included in data analysis.

### METHOD DETAILS

#### Virus production

AAV1-Ef1a-DIO-mCherry and AAVDJ-SYN-DIO-hM3Dq:mCherry DNA plasmids were provided by B. Roth (Addgene #50462 and #44361). AAV1-SYN-DIO-YFP and AAV1-Ef1a-DIO-ChR2:mCherry DNA plasmids were provided by K. Deisseroth (Stanford University). AAVDJ-Ef1a-DIO-GFP:TeTx and AAV1-CBA-DIO-hM4Di:YFP plasmids were constructed by R. Palmiter (University of Washington). AAV1-CBA-DIO-GCaMP6m DNA plasmid was provided by L. Zweifel. AAV refers to adeno-associated virus; DIO refers to double inverted orientation of loxP sites; YFP refers to yellow fluorescent protein. AAV1 serotype viruses were prepared in-house by transfecting HEK cells with each of these plasmids. Viruses were purified by sucrose and CsCl gradient centrifugation steps and re-suspended in 0.1 M phosphate-buffered saline (PBS) at about 10^13^ viral particles/mL. AAVDJ serotype viruses were prepared by Janelia Viral Tools lab.

#### Partial sciatic nerve ligation

Partial sciatic nerve ligation (pSNL) was performed as described.^33^ In brief, mice were anesthetized with 2% isoflurane at a flow rate of 1 L/min. Subsequently a 2-cm incision was made over the lateral aspect of the proximal third of the left hind leg. Blunt dissection was used to visualize the sciatic nerve, which was then exteriorized with hooked forceps. For sham surgeries the sciatic nerve was then immediately returned to its position. For nerve ligation surgeries, a 6-0 silk suture was passed through 30–50% of the nerve bundle, before being tightly ligated and crushed. The skin was then closed with 6-0 silk sutures.

#### Intrathecal injection of NPY

Intrathecal injection of [Leu^31^, Pro^34^]-NPY (TOCRIS) was performed as described.^61^ In brief, a 30G needle attached to a 25 μL syringe (Hamilton) was inserted between the L5/L6 vertebrae, puncturing the dura mater; 5 μL of vehicle (0.9% saline) or NPY was injected. Each mouse was injected twice, once with vehicle and once with NPY, with 48 h between injections.

#### Stereotaxic surgery

Mice were anesthetized with 2% isoflurane at a flow rate of 1 L/min. After anesthesia induction mice were placed on a stereotaxic frame (David Kopf Instruments). Stereotaxic coordinates in the anterior posterior plane were normalized using a correction factor (F = (bregma – Lambda)/4.21). Viral injections were performed bilaterally for all experiments into the PBN (anterior-posterior: −3.1, rostral-caudal: −4.8, medial-lateral: +/−1.3) at a rate of 0.2 μL/min for 2.5 min for a total of 0.5 μL. For optogenetic stimulation experiments, fiberoptic cables were implanted immediately posterior (anterior-posterior: −3.05, rostral-caudal: −4.8, medial-lateral: +/−1.3) to the viral injection site. To secure the lens to the skull, C&B Metabond (Parkell) and dental cement were used. At the end of each experiment animals were euthanized with phenobarbital and the brain was extracted for histological evaluation of viral placement and fiber optic placement.

For Ca^2+^ imaging experiments *in vivo*, viral injections were in the external lateral part of the parabrachial nucleus using the following coordinates relative to bregma at the skull surface: rostral-caudal: −4.90 mm; medial-lateral: ±1.35 mm; anterior-posterior: +3.40. Viruses were injected unilaterally (randomly assigned) 0.5 μL at 0.1 mL/min. A GRIN lens (Inscopix) was positioned above the target area (rostral-caudal: −4.80 mm; medial-lateral: ±1.7 mm; anterior-posterior: +3.65), and three tungsten wires protruding approximately 0.5 mm beyond the lens surface were attached to the lens to reduce motion artifacts during imaging. The lens was then lowered at a rate of 0.1 mm/min. To secure the lens to the skull, C&B Metabond (Parkell) and dental cement were used. After 4 weeks of recovery, mice were tested to ensure field of view (FOV). Animals with a stable FOV were used in the experiments for the subsequent 2–4 weeks.

#### Allodynia and hyperalgesia assays

The von Frey, tactile-sensitivity assay was performed using the ascending application method as described.^33^ Each animal was placed in a 11.5-cm by 7.5-cm chamber with a wire mesh floor. Animals were acclimated to the chamber for 30 min before the assay began. Filaments were applied to the plantar surface of each hind paw a total of 5 times. Filament application began with a 0.16-g filament and ended after two consecutive filaments elicit a paw-withdrawal on 3 or more out of 5 applications. The paw-withdrawal thresholds for each hind paw were measured and averaged because there was no significant difference in the left/right paw-withdrawal thresholds following any of the performed manipulations (pSNL, NTG, LiCl, etc.).

The Hargreaves thermal-sensitivity assay was performed as described.^71^ Each animal was placed in an 11.5-cm by 7.5-cm chamber with a wire mesh floor. Animals were acclimated to the chamber for 30 min before the assay began. Each animal received infrared thermal stimulation (Ugo Basil, model 37370) a total of 3 times on both hind paw plantar surfaces. Latency to paw-withdrawal was averaged across the 3 sessions. The paw-withdrawal latency for each paw was averaged as we did not see a difference in their response latency values.

The hot-plate assay was performed as described.^55^ Each animal was placed in a 16.5-cm by 16.5-cm chamber on a 55°C hot plate for 30 s. Each animal’s total number of nocifensive behaviors on the plate (paw flicks or licks, and jumps) was recorded.

#### Anxiety and depression assays

The open-field assay was performed as described.^31^ Each animal was placed in a 40-cm by 40-cm white plexiglass chamber for 10 min. The sessions were recorded using a USB camera attached to a personal computer. Locomotor and center time data were collected using Ethovision (Noldus).

The elevated-plus-maze assay was performed as described.^31^ The custom-made EPM consisted of two sets of crossed arms (two arms enclosed by 30 cm tall transparent plexiglass, two arms open), each 50-cm long, 8-cm wide, and set 65-cm above the floor. Mice were allowed to explore the arena for 10 min. The sessions were recorded by a USB camera attached to a personal computer and were analyzed using EthoVision (Noldus).

The tail-suspension test was performed by suspending each animal by the tail using laboratory tape for 1 min. The sessions were recorded by a USB camera attached to a personal computer and were manually scored for freezing behavior by a blinded experimenter.

The sucrose-preference test was performed by housing the animals in caging systems adapted to hold two sipper bottles. The animals were water restricted for 12 h prior to the experiment during the dark cycle. After water restriction, they were presented with two sipper bottles, one with water and one with 1% sucrose solution. The total fluid consumption from each sipper bottle over a 2-h access period was measured.

#### Pharmacological injections

Clozapine N-oxide, nitroglycerin, cisplatin and saline or vehicle controls were injected at 10 mL/kg body weight. CNO was administered at 1 mg/kg for hM3Dq experiments and 5 mg/kg for hM4Di experiments, nitroglycerin at 10 mg/kg, cisplatin at 2.3 mg/kg and saline at 0.9% sodium chloride; vehicle for nitroglycerin was 6% propylene glycol 6% ethanol and saline. Lithium chloride (Fisher Scientific) and the saline control for this condition were injected at 15 mL/kg. Lithium chloride was administered at 0.2 M and the saline was 0.9% sodium chloride. All paw-withdrawal measurements were made 2 h after each CNO injection and daily thereafter unless otherwise indicated.

#### Optogenetic stimulation

After recovery from surgery, mice were acclimated to fiber-optic cable attachment. For von Frey, allodynia assessment, light-pulse trains (20 Hz; 2 s “on”, 2 s “off”) were delivered for 5 or 20 min as described in the text. Stimulation paradigms were programmed using a Master8 (AMPI) pulse stimulator that controlled a blue-light laser (473 nm; LaserGlow). The power of light exiting each side of the branching fiber-optic cable was adjusted to 10 ± 1 mW. All paw-withdrawal measurements were made 2 h after each ChR2 stimulation and daily thereafter.

#### Immunohistochemistry

Mice were anesthetized with phenobarbitol (0.2 mL, i.p.) and perfused transcardially with phosphate-buffered saline (PBS) followed by 4% paraformaldehyde (PFA, Electron Microscopy Sciences) in PBS. Brains were post-fixed overnight in 4% PFA at 4°C, cryoprotected in 30% sucrose, frozen in OCT compound and stored at −80°C. Coronal sections (30 μm) were cut on a cryostat (Leica Microsystems) and collected in cold PBS. For immunohistochemistry experiments, sections were washed three times in PBS with 0.2% Triton X-100 (PBST) for 5 min and incubated in blocking solution (3% normal donkey serum in PBST) for 1 h at room temperature. Sections were incubated overnight at 4°C in PBS with primary antibodies including: chicken-*anti*-GFP (1:10000, Abcam, ab 13970) or rabbit-*anti*-dsRed (1:1000, Tacara, ab 632496). After 3 washes in PBS, sections were incubated for 1 h in PBS with secondary antibodies: Alexa Fluor 488 donkey anti-chicken or Alexa Fluor 594 donkey anti-rabbit (1:500, Jackson ImmunoResearch). Tissue was washed 3 times in PBS, mounted onto glass slides, and coverslipped with Fluoromount-G (Southern Biotech). Fluorescent images were acquired using a Keyence BZ-X700 microscope. Images were minimally processed using ImageJ software (NIH) to enhance brightness and contrast for optimal representation of the data. All digital images were processed in the same way between experimental conditions to avoid artificial manipulation between different datasets.

#### RNAscope *in situ* hybridization

Mice were anesthetized with phenobarbital (0.2 mL, i.p.) then decapitated. Brains were rapidly frozen on crushed Dry Ice. Coronal sections (20 μm) were cut on a cryostat (Leica Microsystems), mounted onto glass slides, and stored at −80°C. RNAscope fluorescent multiplex assay was performed following the manufacturer’s protocols. Samples were taken from 2 males and 2 females in each group (3 and 30 day) and several levels of the PBN were imaged for each animal using a Keyence BZ-X710 microscope. Using the superior cerebellar peduncle (scp) as the center point, images were acquired at 20× in a 3 × 3 grid then stacked and stitched together using Fiji for an initial total of 116 PBNs. Images of probe staining within the four-channel sets were subtracted from one another using Fiji’s image calculator function to remove background autofluorescence and minimally processed to enhance brightness and contrast for optimal representation of the data. After image optimization, due to difficulty in getting precisely matching bregma levels during sectioning, PBN anatomy was evaluated using fiber-tract location and general structure to categorize the images into two groups. “Rostral” sections were defined by presence of the caudal part of the nucleus of the lateral lemniscus (NLL) and a more triangular shape of the lateral PBN (bregma −4.95 to −5.15), and “middle” sections were defined by presence of the longer central part of the ventral spinocerebellar tract (sctv) and narrower oval appearance of lateral PBN (bregma 5.15 to −5.35). Sections that were deemed to be further rostral and caudal of these two categories, and sections that had tissue damage in the PBN were removed for a final count of 89 PBN images. Images were imported into QuPath and a region of interest was drawn over the lateral PBN using the surrounding fiber tracts and brain structure as a guide. RNA expression was quantified by thresholding using the subcellular detection function in QuPath. Cells were deemed positive for *Calca* or *Cck* if they had 5 or more puncta and cells were deemed positive for *Fos* if they had 4 or more puncta because *Calca* and *Cck* had denser transcript labeling. An average of ~1700 DAPI-positive cells were analyzed for each section for a total of ~150,000 cells analyzed.

#### Electrophysiology

Mice were deeply anesthetized with Euthasol (i.p. 1 μL per 10 g body weight) and intracardially perfused with ice-cold cutting solution containing (in mM): 92 N-methyl-D-glucamine, 25 D-glucose, 2.5 KCl, 10 MgSO_4_, 1.25 NaH_2_PO_4_, 30 NaHCO_3_, 0.5 CaCl_2_, 20 HEPES, 2 thiourea, 5 Na-ascorbate, 3 Na-pyruvate. Brains were quickly removed after perfusion and 250-μm coronal slices were prepared (Leica VT1200) in the same ice-cold solution. Brain slices were kept in the cutting solution at 33°C for 10 min and then transferred to a room temperature recovery solution containing (in mM): 13 D-glucose, 124 NaCl, 2.5 KCl, 2 MgSO_4_, 1.25 NaH_2_PO_4_, 24 NaHCO_3_, 2 CaCl_2_, 5 HEPES for at least 1 h. Slices were individually transferred to 33°C artificial cerebral spinal fluid containing (in mM): 11 D-glucose, 126 NaCl, 2.5 KCl, 1.2 NaH_2_PO_4_, 26 NaHCO_3_, 2.4 CaCl_2_, 1.2 MgCl_2_ for recording. All solutions were saturated with 95% O2/5% CO2 and adjusted to pH 7.3–7.4, 300–310 mOsm.

Epifluorescence microscope (OLYMPUS BX51WI) was used to visualize *Calca* neurons expressing AAV-DIO-hM3Dq-mCherry and AAV-DIO-mCherry. A 3–5 MΩ glass pipet containing (in mM): 135 K-gluconate, 4 KCl, 10 HEPES, 4 Mg-ATP, 0.3 Na-GTP (pH 7.35, 280–300 mOsm) was used to record neuron activity. To record intrinsic firing frequencies, 800-ms current injections with 20-pA steps from −100 pA to 240 pA were applied in current clamp, with initial holding potential at −70 mV and repeated every 10 s. To record the efficiency of CNO application, cell-attached measurement was used to record action potential in voltage clamp with 0 pA holding current. CNO (3 μM) was bath applied after 3 min of action potential firing. All data were obtained using MultiClamp 700B amplifier (Molecular Devices). Data acquisition and analysis were done using pClamp 11 and Clampfit 11.0.3.

#### Calcium imaging

AAV-Ef1a-DIO-GCaMP6m was injected into the PBN of *Calca*^*Cre/*+^ mice. After 6 weeks of recovery, the nVista (Inscopix) microscope was attached and connected once a week to check the field of view. Recording was conducted using the IDAS program (Inscopix), and the best focus was determined through visual inspection. Occasionally, the mice received a brief air puff as an aversive stimulus. For multi-day imaging of *Calca* neurons, nVista was connected Ethovision (Noldus) via BNC cable to synchronize video recording and Ca^2+^ imaging to ensure that the onset and offset of the Ca^2+^ imaging session matched the video recording. The microendoscope was connected to a commutator (Inscopix) and attached to the baseplate on the mouse, which was then housed in an open-top cage for 4 days to maintain the same field of view throughout the experimental period.

On day 1, a vehicle solution (6% propylene glycol 6% ethanol and saline) was injected intraperitoneally, and the mouse was placed in the von Frey-stimulation chamber for a 45-min acclimation. The imaging session consisted of 5 min of basal activity and 10 min of von Frey stimulation. During the von Frey-stimulation sessions, the mouse was exposed to 8 stimulations of 0.4-g von Frey filament with 1-min, inter-trial intervals. After the imaging session, the LED was turned off, but the microendoscope remained connected, and the mouse was then returned to open-top cage until the start of the next day’s experiment. On day 2, NTG (10 mg/kg, dissolved in 6% propylene glycol 6% ethanol and saline) was injected intraperitoneally. The same imaging session as on day 1 was repeated. On days 3 and 4, the mice were placed in the von Frey-stimulation chamber for imaging as on previous days.

The imaging parameters had insignificant photobleaching, but sufficient fluorescence (LED power, 0.2–0.4; sampling rate 10 Hz). The raw data were processed using IDPS software (ver. 1.9.1, Inscopix). All images acquired over 4 days were concatenated for further analysis. After applying 4X spatial and 2X temporal downsampling, the data underwent spatial bandpass filtering to reduce background noise. Subsequently, motion correction was applied to the images based on a reference frame and region of interest (ROI) within the field of view (FOV). Using IDPS, ΔF/F movies were generated, then PCA/ICA analysis was performed to extract neuronal activities. In cases where there was significant motion or higher background noise, a manual ROI analysis method was employed. To do that, the maximum projection images were used as a reference for spatial information of the neurons, and ROIs were manually drawn based on the borders of the neurons. All neurons analyzed using PCA/ICA and manual ROI methods were visually inspected for each cell, taking into consideration their shape and dynamics, to ensure accuracy.

The outputs of PCA/ICA (ΔF/F) were processed using customized MATLAB code to calculate the *Z* score. In the case of manual ROI analysis, ΔF/F was calculated as ΔF/F=(F–Fmean)/Fmean. Fmean indicates mean fluorescent during day 1 baseline. For basal activity analysis, concatenated Ca^2+^ traces were used to calculate *Z* score, and the Z-scored data were compared across days using the formula: Z=(F–Fmean)/Fstd. Fstd indicates standard deviation of fluorescent during day 1 baseline. For the analysis of von Frey filament-elicited responses, the traces from −20 s to 20 s were extracted and used to calculate the Z-scores. To compare the fluorescent responses, all data points were calculated relative to the −20 s–0 s periods because the mice exhibited different basal activities every day due to intraperitoneal injections or allodynia.

Responsive neurons were identified through statistical analysis comparing the area under the curve (AUC) of pre- and post-stimulation. The AUCs were calculated for three time blocks (−5 to 0 s, 0 to 5 s, and 5 to 10 s) across 8 trials and compared using the Wilcoxon Signed-rank test. Neurons were classified as “increased” if there was a significant increase in the AUC in more than one post-stimulation block. If neither post-stimulation block showed statistical significance, neurons were classified as “unresponsive”. Neurons that exhibited a statistically significant decrease in activity across two blocks were classified as “decreased”.

### QUANTIFICATION AND STATISTICAL ANALYSIS

Data were analyzed in GraphPad Prism 9.5.1 (Graphpad Software) by two-way ANOVA with Šidák post hoc correction; for longitudinal assays Bonferroni’s multiple comparisons correction was applied; p < 0.05 was deemed statistically significant. All data are presented as the mean ± standard error of the mean (SEM). The asterisks in the figures represent the p values of post hoc tests corresponding to the following values *p < 0.05, **p < 0.01, ***p < 0.001, ****p < 0.0001. Following histology and imaging, any mouse whose targeted injection site was wrong was excluded from experimental analysis. Biological and technical replicate n values can be found in figure legends. The assumptions required for our statistical analysis were accounted for in our experimental design, but not specifically tested for post-hoc.

## Supplementary Material

1

## Figures and Tables

**Table:** KEY RESOURCES TABLE

REAGENT or RESOURCE	SOURCE	IDENTIFIER
Antibodies		
Rabbit-anti-dsRed	Takara	ab 632496; RRID: AB_10013483
Chicken-anti-GFP	Abcam	ab 13970; RRID: AB_300798
Alexa Fluor 594 donkey anti-rabbit	Jackson ImmunoResearch	#715-585-150; RRID: AB_2340854
Alexa Fluor 488 donkey anti-chicken	Jackson ImmunoResearch	#703-545-155; RRID: AB_2340375
RNAscope Probe Mm-*Fos*-C1	ACD Biotechne	Ref #316921
RNAscope Probe Mm-*Calca*-C2	ACD Biotechne	Ref #578771-C2
RNAscope Probe Mm-*Cck*-C2	ACD Biotechne	Ref #402271-C3
Bacterial and virus strains		
pAAV1-SYN1-DIO-YFP	Karl Deisseroth	Like Addgene plasmid #27056
pAAVDJ-Ef1a-DIO-GFP:TeTx	Janelia	N/A
pAAV1-Ef1a-DIO-ChR2:mCherry	Mattis et al.^74^	Addgene #35508
pAAVDJ-SYN1-DIO-hM3Dq:mCherry	Krashes et al.^75^	Addgene #44361
pAAV1-Ef1a-DIO-mCherry	Bryan Roth	Addgene #50462
pAAV1-Ef1a-DIO-GCaMP6m	Larry Zweifel	N/A
pAAV1-hSyn-DIO-hM4:mCherry	Krashes et al.^75^	Addgene #44362
Chemicals, peptides, and recombinant proteins		
Clozapine-N-Oxide	Fisher Scientific	Cat #L121, CAS 7447-41-8
Nitroglycerin	American Regent, Inc.	NDC 0517-4810-25
Lithium Chloride	Fisher Scientific	Cat #L121, CAS 7447-41-8
Cisplatin	West-Ward	NDC 0143-9504-01
Paraformaldehyde 32% Aqueous Solution	Electron Microscopy Sciences	Cat #15714-S
Phenobarbitol	Akorn	NDC 59399-185-90
Normal donkey serum	Jackson ImmunoResearch	Cat #017-000-121, RRID: AB_2337258
O.C.T.	Thermofisher	Cat #23-730-571
DAPI Fluoromount-G	SouthernBiotechne	Cat #0100-20
[Leu^31^, Pro^34^]-NPY	Tocris	Cat #1176
C&B Metabond	Parkell	SKU: S380
Euthasol	Virbac	Ref# 200-071
Dental Cement	A-M Systems	Ref# 594845
Critical commercial assays		
RNAscope Fluorescent Multiplex Assay V1	ACD Biotechne	Discontinued
Experimental models: Organisms/strains		
Mouse: C57BL/6J	Jackson Laboratory	JAX:000664
Mouse: *Calca*^*Cre*^	Jackson Laboratory	JAX:033168
Mouse: *Slc17a6*^*Cre*^	Jackson Laboratory	JAX:016963
Mouse: *Oprm1*^*Cre*^	Jackson Laboratory	JAX:035574
Mouse: *Calca*^*tdTomato*^	Pauli et al.^12^	N/A
Software and algorithms		
QuPath	GitHub	https://qupath.github.io/
Prism (v9.5.1)	GraphPad Software	www.graphpad.com
Fiji	Schindelin et al.^76^	www.fiji.sc
ImageJ	Schneider et al.^77^	www.imagej.net
pClamp 11	Molecular Devices	www.moleculardevices.com
Clampfit 11.0.3	Molecular Devices	www.moleculardevices.com
IDAS	Incsopix	www.inscopix.com
IDPS	Inscopix	www.inscopix.com
Original Code	This study	https://doi.org/10.5281/zenodo.10725342
MATLAB	Mathworks	www.mathworks.com/products/matlab.html
Ethovision	Noldus Technology	www.noldus.com
Other		
Modular chambers and elevated mesh bottom stand	Bioseb	Ref #BIO-PVF
Plantar test for thermal stimulation	Ugo Basil	Product code: 37370
Hot plate	Bioseb	Ref #BIO-CHP
GRIN Lens	Inscopix	www.inscopix.com
Cryostat	Leica	Ref# CM1950
Fluorescence Microscope	Keyence	Ref# BZ-X700
473 nm Laser	LaserGlow	Ref# LRS-0473
Epifluorescence Microscope	Olympus	Ref# BX51WI
Microelectrode Amplifier	Molecular Devices	SKU: MultiClamp 700B
6-0 Silk Suture	ProAdvantage	SKU: PROA-P420683
Stereotaxic Frame	David Kopf Instruments	Model 1900
Master-8 Pulse Stimulator	A.M.P.I.	N/A
Keyence All-in-One Fluorescence Microscope	Keyence	Ref# BZ-X710
Fiberoptic Cables	Doric Lenses	Ref# MFP_200
Vibratome	Leica	Ref# VT1200
nVista Microscope	Inscopix	www.inscopix.com
Commutator	Inscopix	www.inscopix.com

## Highlights

- Neuropathic injury chronically increases the intrinsic excitability of *Calca* neurons
- *Calca* neurons are necessary for the manifestation and maintenance of chronic pain
- Activity within *Calca* neurons is sufficient to induce nociplasticity
- Aversive stimuli of many sensory modalities can induce chronic pain via *Calca* neurons
