# Supplementary material for: Parabrachial Calca neurons drive nociplasticity
Source: Cell Rep. Author manuscript; Available in PMC 2024 Jun 27. (PMC11210282; doi:10.1016/j.celrep.2024.114057)
Supplement: 1 [file NIHMS1989485-supplement-1.pdf]

Cell Reports, Volume *43*

Supplemental information

Parabrachial *Calca* neurons drive nociplasticity

Logan F. Condon, Ying Yu, Sekun Park, Feng Cao, Jordan L. Pauli, Tyler S. Nelson, and Richard D. Palmiter

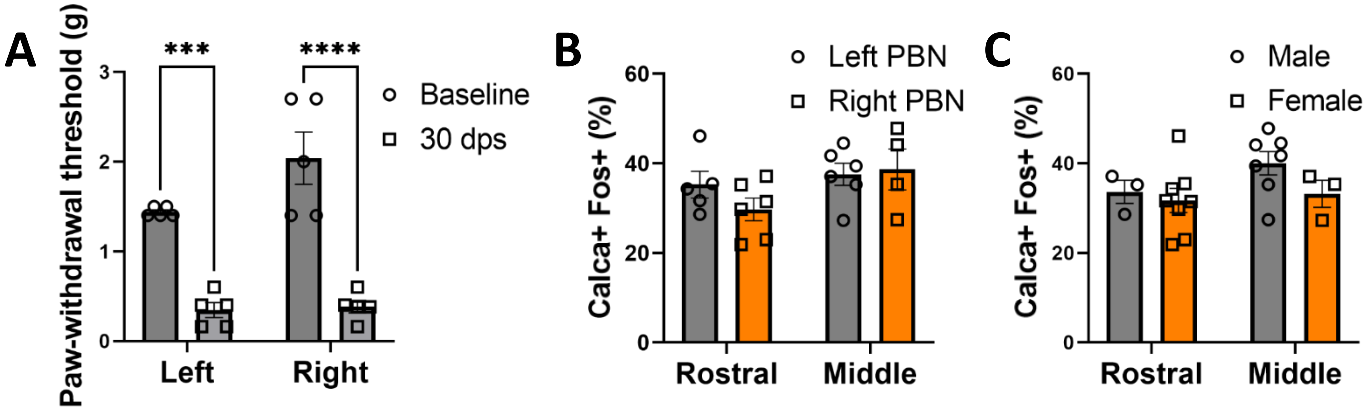

Figure S1. PBN *Calca* neurons bilaterally exhibit uniform activity and do not have sexual dimorphic activity. (Related to Figure 1)

(A) pSNL resulted in bilateral allodynia, n = 5.
(B) There was no difference between left and right PBN *Calca* and *Fos* colocalization 3 days post unilateral (left) pSNL. Rostral left PBN, n = 5; rostral right PBN, n = 6; middle left PBN, n = 6; middle right PBN, n = 4.
(C) There was no difference between male and female PBN *Calca* and *Fos* colocalization 3 days post pSNL. Rostral male, n = 3; rostral female, n = 7; middle male, n = 7; middle female, n = 3.

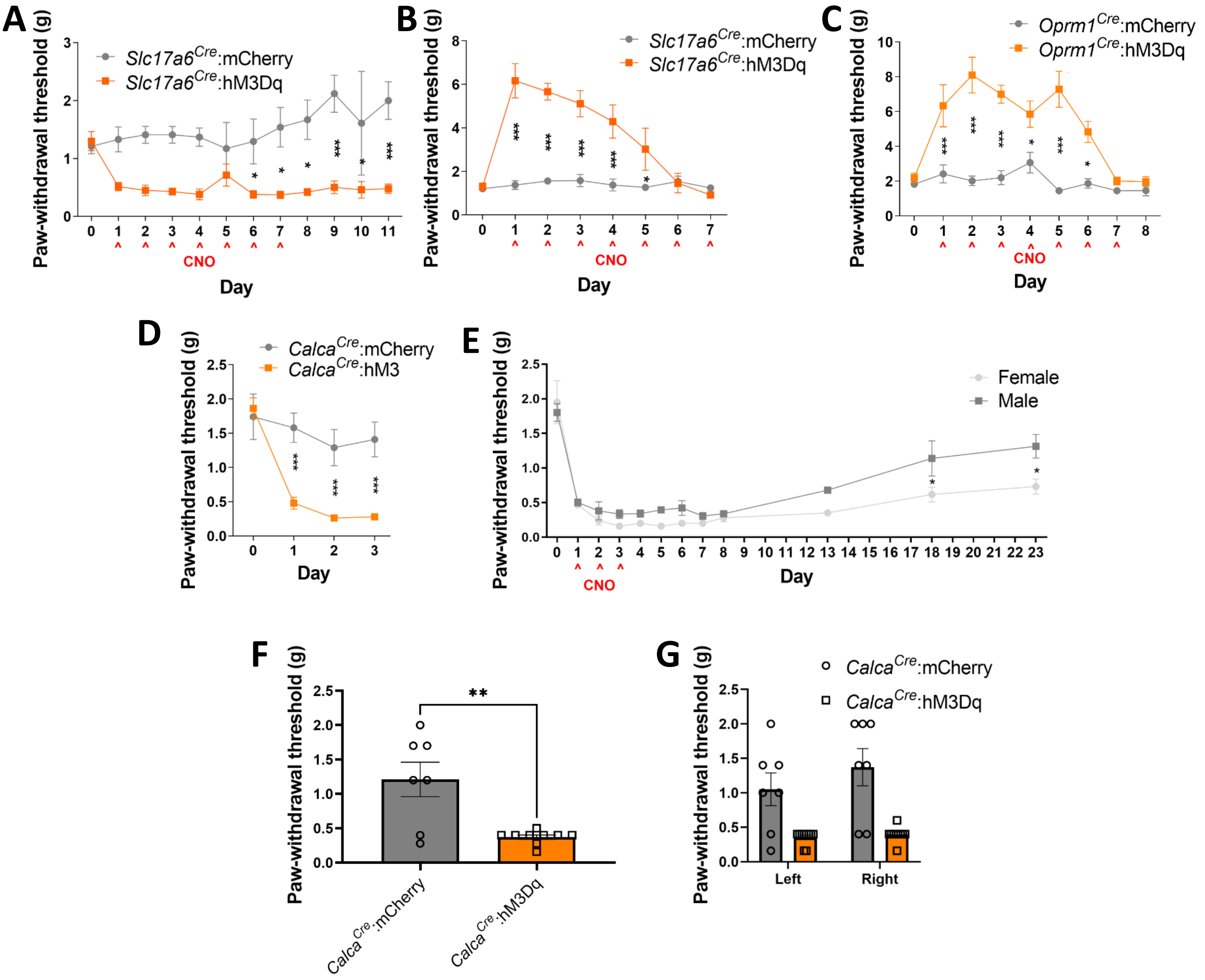

Figure S2. Stimulation of PBN subpopulations (Related to Figure 3)

(A) Chronic stimulation of PBN *Slc17a6* (Vglut 2) neurons via hM3Dq and CNO resulted in persistent allodynia (measured 23 h post injection).
(B) Stimulation of PBN *Slc17a6* neurons via hM3Dq and CNO resulted in analgesia 2 h post injection.
(A-B) *Slc17a6*^{Cre}:mCherry n = 5, *Slc17a6*^{Cre}:hM3Dq n = 6.
(C) Stimulation of PBN *Oprm1* neurons via hM3Dq and CNO resulted in analgesia 2 h post injection. *Oprm1*^{Cre}:mCherry n = 5, *Oprm1*^{Cre}:hM3Dq n = 7.
(D) Stimulation of PBN *Calca* neurons via hM3Dq and CNO resulted in allodynia 23 h after CNO treatment. *Calca*$^{cre/+}$:mCherry n = 5 and *Calca*$^{cre/+}$:hM3Dq n = 7.
(E) Stimulation of PBN *Calca* neurons via hM3Dq and CNO resulted in persistent allodynia in both male and female animals. Female, n = 3; male, n = 4.
(F) Unilateral stimulation of PBN Calca neurons via hM3Dq and one injection of CNO resulted in allodynia measured 2 hr later. *Calca*$^{cre/+}$:mCherry n = 7 and *Calca*$^{cre/+}$:hM3Dq n = 10.
(G) Unilateral stimulation of PBN Calca neurons via hM3Dq and CNO affected left and right hind paw-withdrawal threshold equivalently. *Calca*$^{cre/+}$:mCherry n = 7 and *Calca*$^{cre/+}$:hM3Dq n = 10.

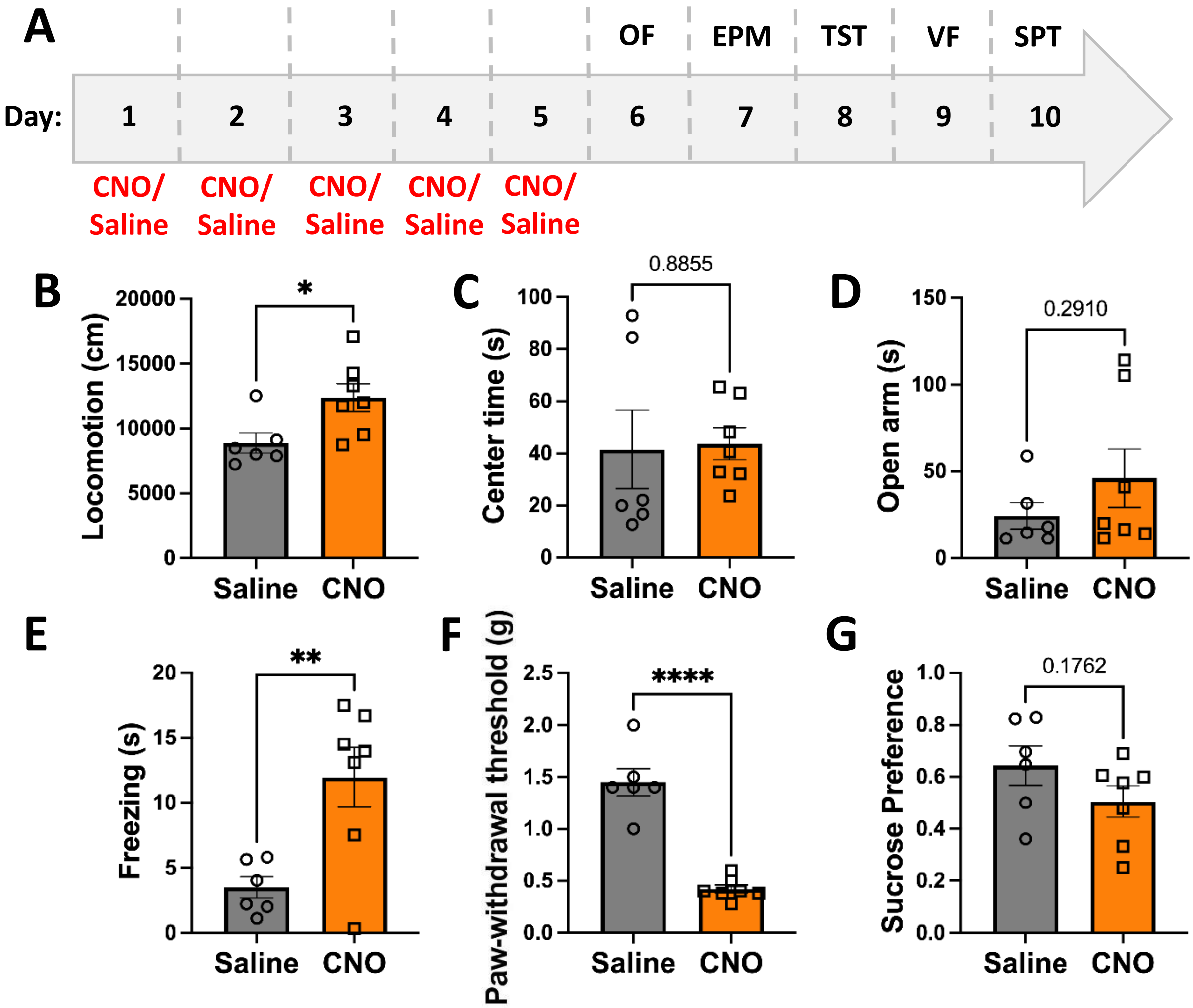

Figure S3. Chronic stimulation of PBN subpopulations (Related to Figure 3)

(A) Behavior timeline for open field (OF), elevated plus maze (EPM), tail-suspension test (TST), von Frey (VF), and sucrose-preference test (SPT) before and after 5 consecutive days of CNO or saline injection. *Calca*$^{Cre/+}$:hM3Dq with i.p. saline n = 6 and *Calca*$^{cre/+}$:hM3Dq with i.p. CNO n = 6.
(B) 5 days of CNO injection increased open field locomotion.
(C) 5 days of CNO injection did not affect time spent in the center of the arena during the open field assaay.
(D) 5 days of CNO injection did not affect time spent in the open arms during the elevated plus maze assay.
(E) 5 days of CNO injection increased time spent freezing during the tail suspension test.
(F) 5 days of CNO injection decreased paw-withdrawal threshold in the von Frey assay.
(G) 5 days of CNO injection did not significantly affect sucrose preference in the sucrose preference test.

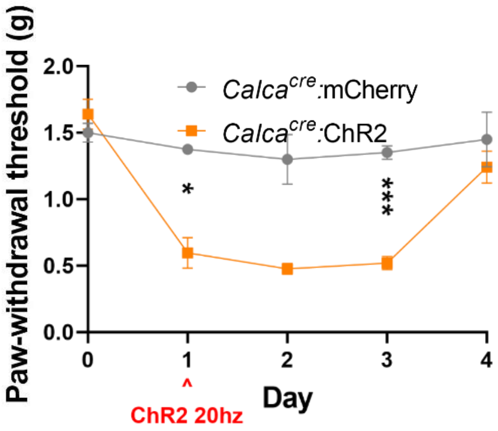

Figure S4. Unilateral optogenetic stimulation of *Calca* neurons (Related to Figure 4)

Unilateral stimulation of PBN *Calca* neurons via ChR2 and 473-nm light (20 min, 20 Hz, 2 s on 2 s off) resulted in persistent allodynia. *Calca*$^{cre/+}$:mCherry n = 4 and *Calca*$^{cre/+}$:ChR2 n = 4.

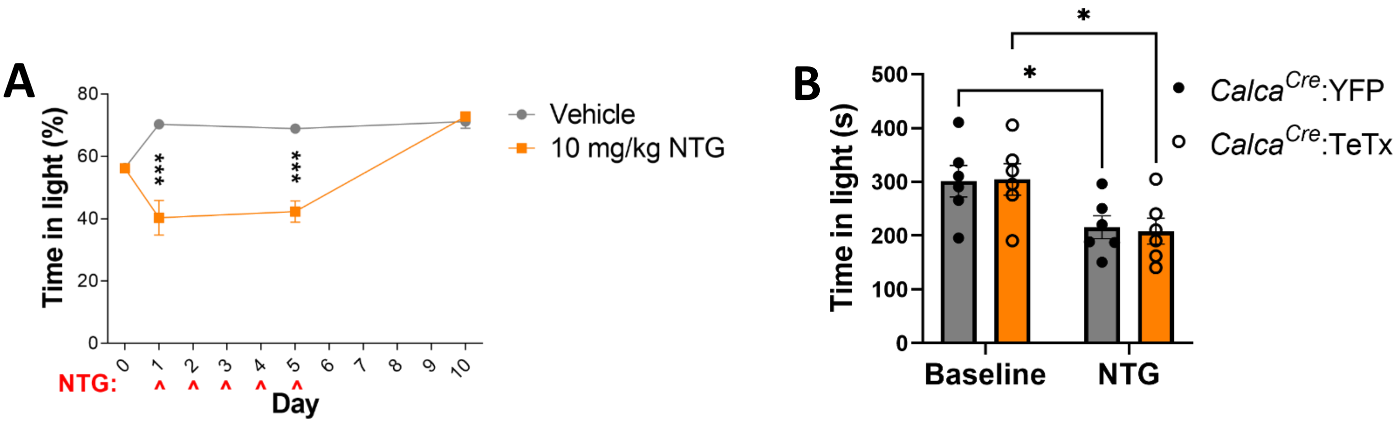

Figure S5. NTG induced photophobia (Related to Figure 5)

(A) NTG injection resulted in photophobia that did not persist past the point of NTG administration. Vehicle, n = 4; NTG, n = 4.
(B) TeTx expression in PBN *Calca* neurons did not prevent the development of NTG-driven photophobia. *Calca*$^{Cre/+}$:YFP n = 6 and *Calca*$^{Cre/+}$:TeTx n = 6.

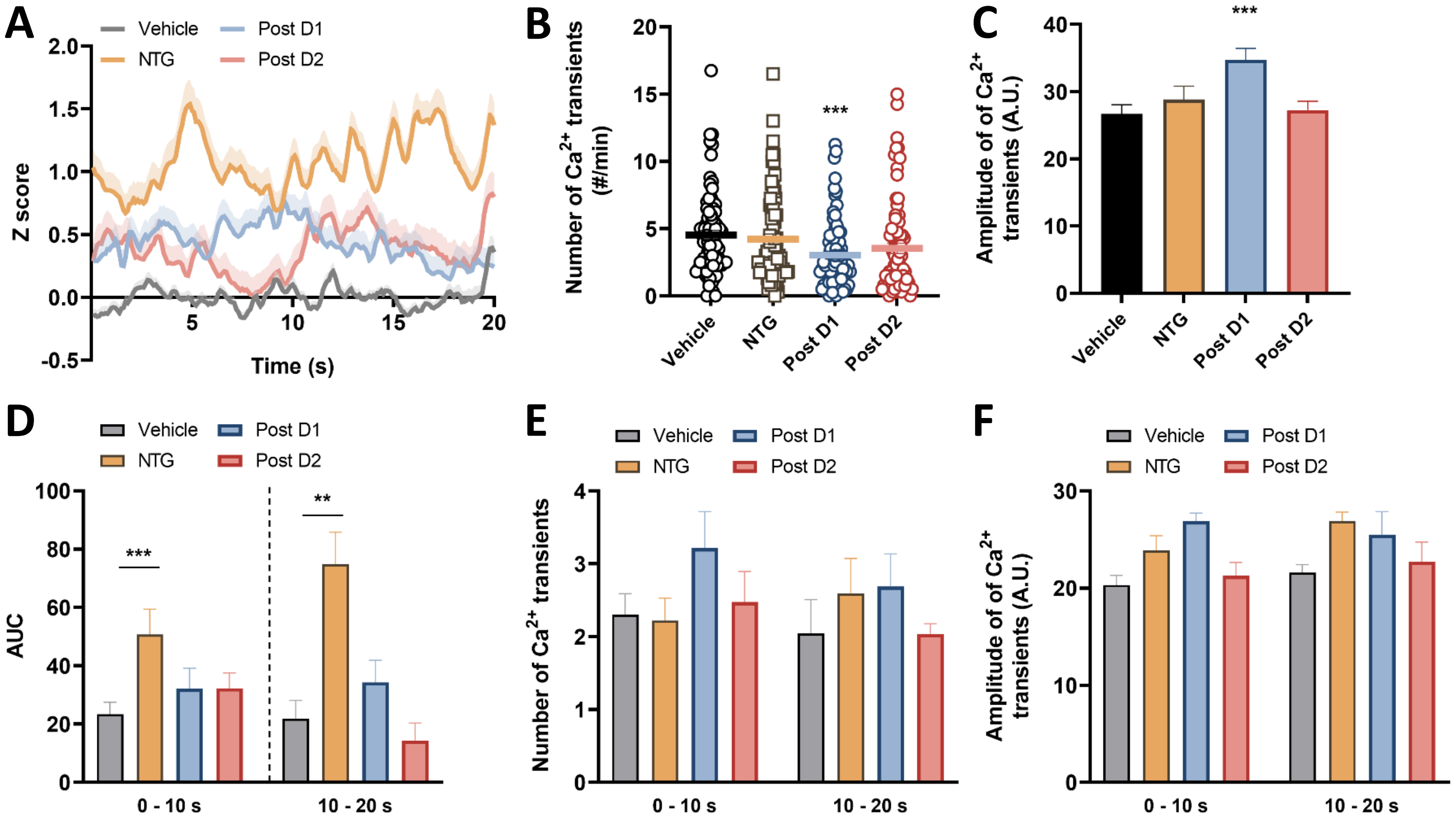

Figure S6. NTG injection does not affect the total number of or amplitude of calcium transients in *Calca* neurons. (Related to Figure 6)

(A) Average traces of individual neurons during baseline period (10 min before von Frey filament application). Shaded area indicates. +S.E.M.
(B) Number of Ca2+ transients during 10 min of baseline.
(C) Amplitude of Ca2+ transients during 10 min of baseline.
(D) Average AUC of individual neurons evoked by von Frey filament. Bar indicates mean S.E.M.
(E) Number of Ca2+ transients during 10 min of baseline.
(F) Peak amplitude during 20 sec of post stimulation period.
(A-F) n = 3 animals, 79 neurons.
